# A gene subset requires CTCF bookmarking during the fast post‐mitotic reactivation of mouse ES cells

**DOI:** 10.15252/embr.202256075

**Published:** 2022-11-04

**Authors:** Almira Chervova, Nicola Festuccia, Luis Altamirano‐Pacheco, Agnès Dubois, Pablo Navarro

**Affiliations:** ^1^ Department of Developmental and Stem Cell Biology Institut Pasteur, Université Paris Cité, CNRS UMR3738, Epigenomics, Proliferation, and the Identity of Cells Unit Paris France; ^2^ Equipe Labélisée Ligue Contre le Cancer Paris France; ^3^ Collège Doctoral Sorbonne Université Paris France

**Keywords:** CTCF, ES cells, gene reactivation, hyper‐transcription, mitotic bookmarking, Cell Cycle, Chromatin, Transcription & Genomics, Stem Cells & Regenerative Medicine

## Abstract

Mitosis leads to global downregulation of transcription that then needs to be efficiently resumed. In somatic cells, this is mediated by a transient hyper‐active state that first reactivates housekeeping and then cell identity genes. Here, we show that mouse embryonic stem cells, which display rapid cell cycles and spend little time in G1, also display accelerated reactivation dynamics. This uniquely fast global reactivation lacks specificity towards functional gene families, enabling the restoration of all regulatory functions before DNA replication. Genes displaying the fastest reactivation are bound by CTCF, a mitotic bookmarking transcription factor. In spite of this, the post‐mitotic global burst is robust and largely insensitive to CTCF depletion. There are, however, around 350 genes that respond to CTCF depletion rapidly after mitotic exit. Remarkably, these are characterised by promoter‐proximal mitotic bookmarking by CTCF. We propose that the structure of the cell cycle imposes distinct constrains to post‐mitotic gene reactivation dynamics in different cell types, via mechanisms that are yet to be identified but that can be modulated by mitotic bookmarking factors.

## Graphical abstract

Mouse ES cells exhibit fast global reactivation of their genes following mitosis to restore their regulatory functions before DNA replication. Around 350 genes require the mitotic bookmarking factor CTCF for this process.

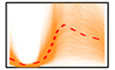

## Introduction

The duration of the cell cycle is highly variable between cell types and during developmental and physiological stages (Dawson *et al*, 1965). A significant amount of this variability depends on the length of the G1 phase (Smith & Martin, 1973), the period following mitosis during which the cell prepares for replicating its DNA and undergoing, or not, an additional division. This transition, known as the G1/S checkpoint, is controlled by extrinsic signals, such as growth factors, which enable progression through the remaining phases of the cell cycle when the environment is favourable for cell growth (Pardee, 1974). The gap between mitosis and replication also provides time for the cell to reinstate an appropriate gene transcription profile. Indeed, mitosis is accompanied by major molecular modifications that entail a drastic downregulation of transcription and a halt of gene regulatory processes (Festuccia *et al*, 2017a). Hence, following mitosis, the cell needs to reactivate its regulatory networks and gene expression to ensure housekeeping functions such as DNA replication or protein synthesis and to maintain the specific molecular and physiological features that define its identity. How the genome undergoes this reawakening of transcription is not entirely understood. Recently, several studies have addressed this question in somatic cells (Hsiung *et al*, 2016; Palozola *et al*, 2017; Kang *et al*, 2020). A picture has emerged that a strong burst of transcription characterises around half of active genes, which may reach after mitosis the highest levels of transcription they will ever attain during the cell cycle (Hsiung *et al*, 2016). Such hyper‐activation is observed first for genes required to rebuild the interphasic cell and only later for those linked to cell identity (Palozola *et al*, 2017; Kang *et al*, 2020). In these three examples studied with sufficient time resolution, the vast majority of genes reach maximal transcription levels during a window of 80–300 min after release from a mitotic block, with the first signs of transcription apparent after approximately 60. Such kinetics are relatively rapid, with respect to the long cell cycles of most cell types, which typically last 24 h. Yet, ensuring the timely reactivation of the genome might be challenging for rapidly proliferating cells. In these cell types, a short G1 phase of less than 2–3 h might require replication to start even if transcription is not fully re‐established. Alternatively, fast cycling cells might display accelerated reactivation dynamics. In support, transformed cells (Palozola *et al*, 2017; Kang *et al*, 2020) appear to reawaken their genome slightly faster than non‐transformed ones (Hsiung *et al*, 2016), suggesting that cellular differences influence the pace of post‐mitotic events. To understand if this relationship applies also to untransformed cells, we focused on undifferentiated mouse Embryonic Stem (ES) cells. Derived from early embryos, these pluripotent cells have an intrinsic demand for fast reactivation after mitosis (Festuccia *et al*, 2017b; Zaveri & Dhawan, 2018), owing to four properties. First, their average cell cycle time is of only 12 h, due to the absence of a G1/S checkpoint; almost immediately after mitosis, ES cells start DNA replication. Second, they self‐renew indefinitely, indicating that they are particularly efficient in maintaining their transcriptome across virtually infinite cell divisions. Third, the preservation of their properties strictly relies on a nearly continuous regulation of gene expression by transcription factors (TFs). Fourth, ES cells were shown to be particularly prone to differentiate after mitosis, indicating that their identity is unstable in G1. We observe that most genes, around 90%, reach maximal transcription levels within the first 2 h after division and a substantial fraction initiate their reactivation as soon as 20–30 min after release from a mitotic block. These kinetics allow the near complete re‐establishment of the transcriptome before the onset of replication. Given that the TF CTCF (Merkenschlager & Nora, 2016) remains bound to a large fraction of its target sites during mitosis (Owens *et al*, 2019), a process known as mitotic bookmarking (Festuccia *et al*, 2017a) and that is either strongly reduced or completely abrogated in other cell types (Oomen *et al*, 2019; Owens *et al*, 2019; Zhang *et al*, 2021a), we hypothesised that CTCF may contribute to the particularly fast and global transcriptional bursting occurring in post‐mitotic ES cells. Using auxin‐inducible depletion of CTCF (Nora *et al*, 2017) we rule out such global function for CTCF, highlighting the robustness of the post‐mitotic burst in ES cells. Despite this, however, we identify around 350 genes enriched in promoter‐proximal mitotic bookmarking by CTCF that display early post‐mitotic responsiveness to CTCF, providing direct experimental evidence of the function of CTCF at the mitosis to G1 transition. We propose that while yet to identify mechanisms may underlie important cell type specificities of the post‐mitotic hyper‐active state, mitotic bookmarking factors play an important role in modulating its dynamics and amplitude for selected groups of target genes.

## Results

### Following post‐mitotic transcription in ES cells with high temporal resolution

**Figure 1:**
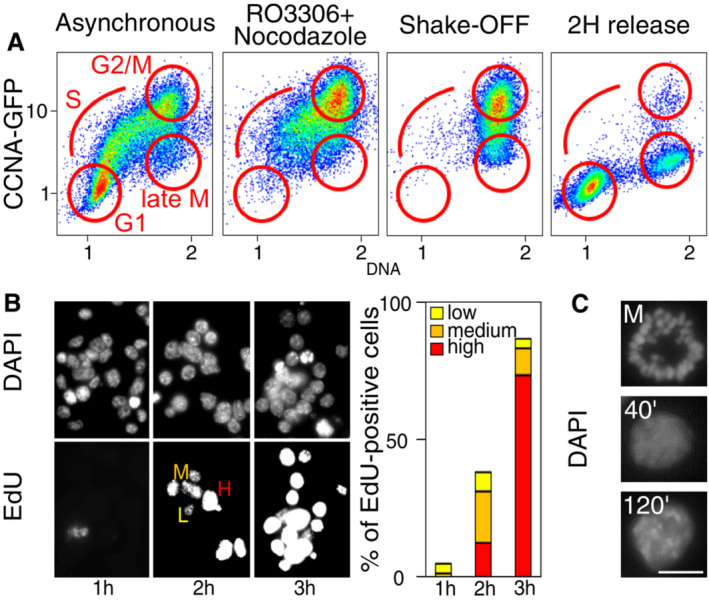
Following gene reactivation in post‐mitotic ES cells with high temporal resolution FACS scatter plots of CCNA‐GFP cell‐cycle reporters (*Y*‐axis, CCNA‐GFP fluorescence, arbitrary units, log scale; *X*‐axis, relative DNA content measured by Hoechst staining). Four conditions are presented, as shown above each panel. Cell cycle phases are indicated in red in the plots.Representative images of cells released from mitosis for 1, 2 or 3 h in EdU‐containing medium. Top, DNA staining with DAPI; Bottom, EdU immunofluorescence. L, M and H indicate cells with low, medium or high levels of EdU incorporation. The barplot shows the quantification of the proportion of replicating cells at each time point.Representative image of cells stained with DAPI upon mitotic arrest and shake‐off (top), and after 40 and 120 min post‐release (middle and bottom). Note obvious chromocentres (DAPI‐dense regions) after 120 min, indicating the restoration of a chromatin organisation typical of interphase. Scale bar: 10 μm.

**Figure 2:**
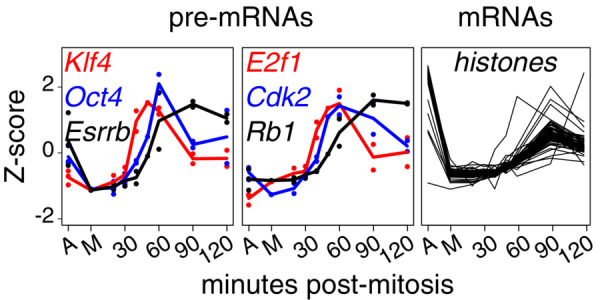
Examples of post‐mitotic gene reactivation *Y*‐axis, *z*‐score; *X*‐axis, timepoints. A indicates asynchronous cells, M mitotic cells and the numbers represent the minutes after release from the mitotic block. Dots show individual replicates and lines averages (*n* = 3 for A and M; *n* = 2 for each post‐mitotic sample). Pre‐mRNA levels are shown for pluripotency genes (*Oct4*, *Klf4*, *Esrrb*) and cell cycle regulators (*E2f1*, *Cdk2*, *Rb*); mRNA levels for individual histone genes (only averages are shown).

We established experimental conditions to follow gene reactivation after mitosis using CCNA‐GFP cell cycle reporters (Festuccia *et al*, 2016) and a double inhibition protocol that allows the efficient block of ES cells in metaphase (4 h CDK1 inhibition with RO‐3306 followed by a release into nocodazole‐supplemented medium for 4 h and shake‐off; Fig 1A). We monitored the dynamics of GFP expression and changes in DNA content by Fluorescent‐Activated Cell Sorting (FACS), as mitotic cells completed division and re‐entered interphase. Two hours post‐release, we observed that a substantial fraction of cells had repopulated the G1 compartment (Fig 1A). We also monitored entry into the S phase by EdU labelling and observed that the vast majority of cells had started DNA replication before 3 h post‐replication (Fig 1B). Moreover, we also observed a gradual decompaction of the chromatin and the acquisition of an interphase‐like nuclear morphology, characterised by prominent chromocentres, during the first 2 h of release (Fig 1C). Therefore, we aimed at analysing gene reactivation during the first 2 h after mitosis. Other studies have used direct methods to monitor post‐mitotic transcription (Hsiung *et al*, 2016; Palozola *et al*, 2017; Kang *et al*, 2020; Pelham‐Webb *et al*, 2021), such as RNAPII profiling or labelling and purification of nascent transcripts in FACS‐purified G1 cells; however, these strategies practically preclude highly resolutive temporal analyses as they require extensive amounts of starting material or manipulation. Hence, in order to generate gene reactivation profiles with high temporal resolution, we decided to use ribo‐depleted, single‐stranded RNA‐seq and a STAR‐RSEM pipeline (Li & Dewey, 2011; Dobin *et al*, 2013) that allowed to quantify intron‐containing pre‐mRNA isoforms. Asynchronous cells were harvested in parallel with mitotic cells and samples obtained at different times following nocodazole release (20, 30, 40, 50, 60, 90 and 120 min) and immediately processed for RNA extraction. Therefore, in comparison to previous studies in ES cells that relied on a maximum of two time‐points after mitosis (Festuccia *et al*, 2016, 2019; Teves *et al*, 2018; Pelham‐Webb *et al*, 2021), our strategy provides an unprecedented temporal resolution, revealing a previously overlooked complexity. For instance, based on an early (1 h post‐mitosis) versus late‐G1 (3 h) analysis, pluripotency‐associated genes have been shown to reactivate fast and remain highly transcribed (Pelham‐Webb *et al*, 2021). However, our time‐resolved analysis shows a variety of behaviours within the first 2 h of release (Appendix Fig S1). Genes such as *Oct4* and *Klf4* display a strong burst at slightly different timepoints, followed by a downregulation; others, like *Esrrb*, reactivate later but remain stably transcribed (Fig 2). Similarly, cell‐cycle associated genes have been previously found to reactivate early or late after mitosis, depending on their function during the G1/S or G2/M transition (Pelham‐Webb *et al*, 2021). Instead, our results show a large variability in gene transcription patterns (Appendix Fig S1), with many cell‐cycle genes showing intense, non‐synchronous early bursts of transcription that may, or may not, be subsequently maintained (Appendix Fig S1 and Fig 2). Therefore, without the required temporal resolution, the differential reactivation kinetics characterising individual genes remains unnoticed (Fig 2). As a final control of the quality of our dataset, we monitored transcription of histone genes, which is known to occur specifically during S phase (Gunjan *et al*, 2005). Accordingly, and in contrast to most other genes (Appendix Fig S1), histones displayed much higher transcriptional levels in asynchronous cells than after division, reflecting a prevalence of cells in S phase in cycling ES cells (~60–70%). Nevertheless, all histone genes showed signs of substantial transcriptional reactivation as soon as 50′ after release (Fig 2), matching a remarkably fast entry into S phase, which could be observed as early as 1 h after division (Fig 1B). Hence, previous studies using 1 h as the first time point after mitosis may have missed early dynamics, which, presumably, are those most tightly linked to processes such as mitotic bookmarking. Thus, our approach captures the kinetics of transcription reactivation linking mitosis to the onset of the S phase with unprecedented details.

### Rapid genome‐wide transcriptional bursting in post‐mitotic ES cells

**Figure 3:**
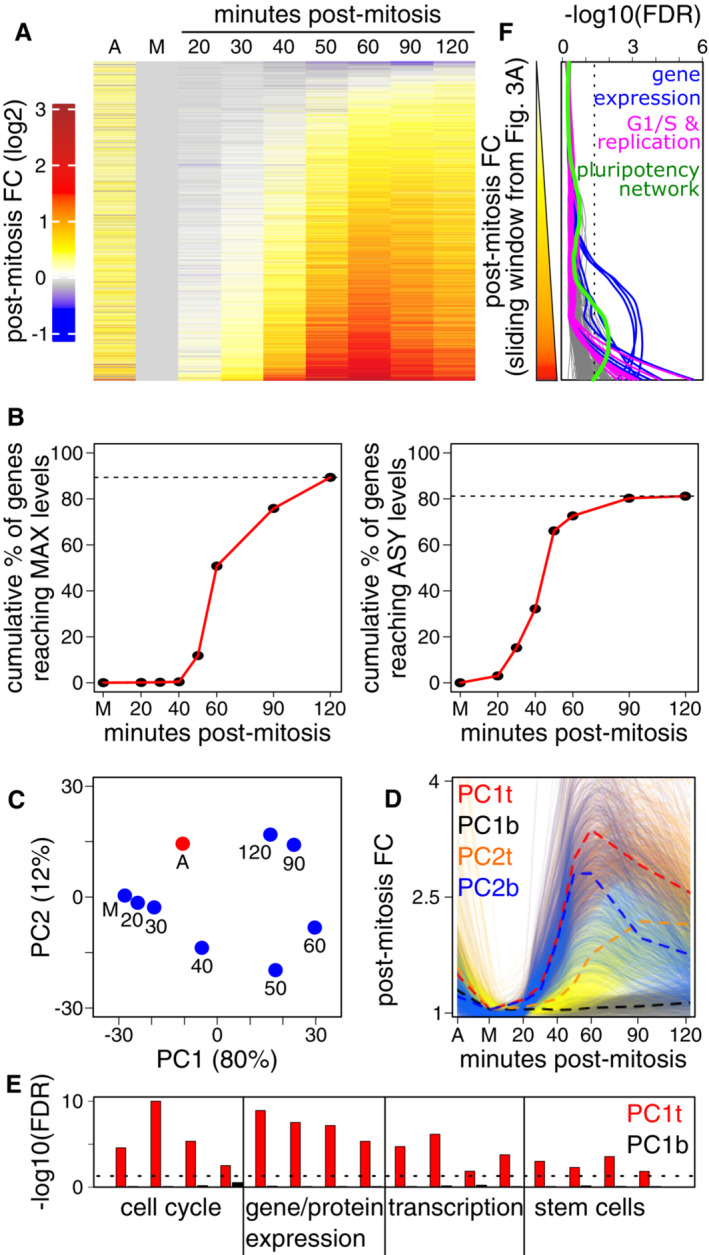
A prominent and fast burst of transcription shortly after mitosis Heatmap showing average log2 fold change to mitosis of pre‐mRNA levels for 9,911 individual genes in asynchronous cells (A), mitotic cells (M), and at each timepoint post release, ranked from top to bottom by the mean of 20–90 min. Two to three independent biological replicates were used, see Materials and Methods for details.Cumulative plots showing the percentage of genes reaching maximal detection levels (left) or the levels detected in asynchronous cells (right) as a function of time. The dashed horizontal lines denote the maximal observed cumulative proportion of genes. Note that around 10% of genes (1,033) display maximal transcription in asynchronous cells. An additional ~10% of genes (831) showed instead low levels of transcription in asynchronous cells compared to mitosis.Principal Component Analysis of all considered samples. The variance captured by each dimension is indicated. Samples are labelled as in Fig 3A.Individual traces (light lines; smoothed with a loess regression) and mean profiles (dashed bold lines) of 1,000 genes identified from the top and bottom PCA loadings of PC1 and PC2, revealing the major tendencies driving post‐mitotic gene reactivation (*Y*‐axis, fold change to mitosis; *X*‐axis as in Fig 2). These four gene lists were subject to gene Ontology analyses, as shown in Appendix Fig S3.Barplot showing −log10(FDR) of selected Terms over PC1t (red) and PC1b (black) gene groups. Each bar corresponds, from left to right, to the cell cycle and replication (GO:0007346, GO:0006261, GO:0000082, GO:0000086), gene/protein expression (GO:0010467, GO:0006397, GO:0042254, GO:0045047), transcription (GO:0045893, GO:0065004, GO:0006354, GO:0000083), stem cells (PluriNetWork WP1763, GO:2000736, GO:0060071, GO:0000165). The dotted line denotes −log10(0.05).Plot showing the −log10(FDR) for the enrichment of a variety of gene ontology terms (Dataset EV2) in groups of 500 genes, defined by sliding a window over genes ranked based on their reactivation kinetics, as in Fig 3A (top, weak post‐mitotic reactivation; bottom, strong post‐mitotic reactivation). The ontology terms plotted are those enriched in the four groups of genes defined in Fig 3D. Note that all categories of terms show increased enrichment for gene groups displaying strong reactivation dynamics after mitosis. Illustrative categories are coloured: Gene/Protein Expression in blue (GO:0006397, GO:0000398, GO:0000377, GO:0010467, GO:0042254, GO:0006412, among others), the G1/S transition and replication in magenta (GO:0000082, GO:0006261, GO:0036388, GO:0006270, among others) and the Pluripotency Network in green as defined in WikiPathways (PluriNetWork WP1763). The dotted line denotes −log10(0.05).

We then aimed at studying the global dynamics of gene reactivation. For this, we focused on around 10,000 Refseq curated genes displaying at least 0.5 pre‐mRNA Transcripts Per Million (TPM) in either asynchronous cells or in two or more samples after nocodazole release (Dataset EV1). These were then ranked by their mean post‐mitosis fold‐change between 20 and 90′ of release, which orders them according to both the timing and the amplitude of their reactivation (Appendix Fig S2A). We observed a fast and global reactivation of the transcriptome, starting at 20–40′ for most genes, reaching particularly high levels within an hour and declining thereafter (Fig 3A). To more directly evaluate the global reactivation dynamics, we computed the proportion of genes reaching either maximal levels (Fig 3B left) or the levels measured in asynchronous cells (Fig 3B right), at each time point. We observed that around 90% of genes reach their maximal transcriptional level earlier than 2 h following mitosis and around 30, 65 and 80% reached or surpassed the levels measured in asynchronous cells at 40, 60 and 120′, respectively. Since this intense burst of transcriptional activity has been previously observed (Hsiung *et al*, 2016; Palozola *et al*, 2017; Kang *et al*, 2020), we aimed at directly comparing these results with previous datasets, even though intrinsic experimental differences may not allow to reach definitive conclusions. Our comparisons indicate that in ES cells this phenomenon occurs faster and the magnitude and the number of genes engaged appear to be substantially higher (Appendix Fig S2B). Moreover, this analysis also identified around 10% of genes that displayed low levels in asynchronous cells but a strong burst after mitosis, possibly reflecting genes with preferential transcription during G1. Principal component analysis (PCA; Fig 3C) confirmed that major global changes take place between 40 and 60′ after mitosis. Following this, the transcriptome became increasingly similar to that of asynchronous cells. Notably, the most pronounced differences to asynchronous cells were observed after 50–60′, coinciding with the maximal transcriptional peak. PC1 and PC2 capture ~80 and ~12% of the measured variance, respectively. Therefore, we selected the top and bottom 1,000 genes contributing to PC1 and PC2 (PC1t, PC1b, PC2t, PC2b in Fig 3D) corresponding to four major dynamics contributing to the temporal changes of the transcriptome: PC1t and PC2t genes are detected at high levels in asynchronous cells and are either rapidly (PC1t, in red) or more slowly reactivated after mitosis (PC2t, in orange); PC1b and PC2b genes are detected at lower levels in asynchronous cells and either remain poorly transcribed (PC1b, in black) or display a strong and transient transcriptional burst after mitosis (PC2b, in blue). Gene Ontology analyses with these four groups of 1,000 genes characterised by different transcriptional behaviours revealed that those contributing to PC1t are prominently enriched in global cellular functions, the G1/S cell cycle transition and replication, but strikingly also in stem cell–related terms including the pluripotency network (Fig 3E and Appendix Fig S3). This suggests that, in contrast to other cell types (Palozola *et al*, 2017; Kang *et al*, 2020), ES cells rapidly reactivate both cell‐type‐specific genes and genes executing general functions. To test this more thoroughly, we selected all significant associations (FDR < 0.05) derived from the analysis of top/bottom PCA genes (Fig 3D and Dataset EV2) and calculated the FDR corresponding to their enrichment along a sliding window of 500 genes displaying increasingly pronounced and fast reactivation dynamics (from top to bottom in Fig 3A, and see Appendix Fig S2A). This analysis revealed that all terms are strongly biased towards the fastest genes (Fig 3F), including global gene expression functions, the G1/S transition and DNA replication, as well as the pluripotency network. Altogether, we conclude that ES cells display less functional specificity regarding how the genome awakens after mitosis, compared with other cell types where genes associated with the rebuilding of the interphasic cell precede cell type‐specific genes (Hsiung *et al*, 2016; Palozola *et al*, 2017; Kang *et al*, 2020). The nearly simultaneous and fast reactivation of the cell cycle machinery, basal gene expression functions and the pluripotency network in ES cells may enable these cells to cope with a short cell cycle, with the lack of a G1/S checkpoint and satisfy their continued dependency on TFs to self‐renew (Festuccia *et al*, 2017b).

### Proximal CTCF binding correlates with the kinetics of post‐mitotic gene transcription

**Figure 4:**
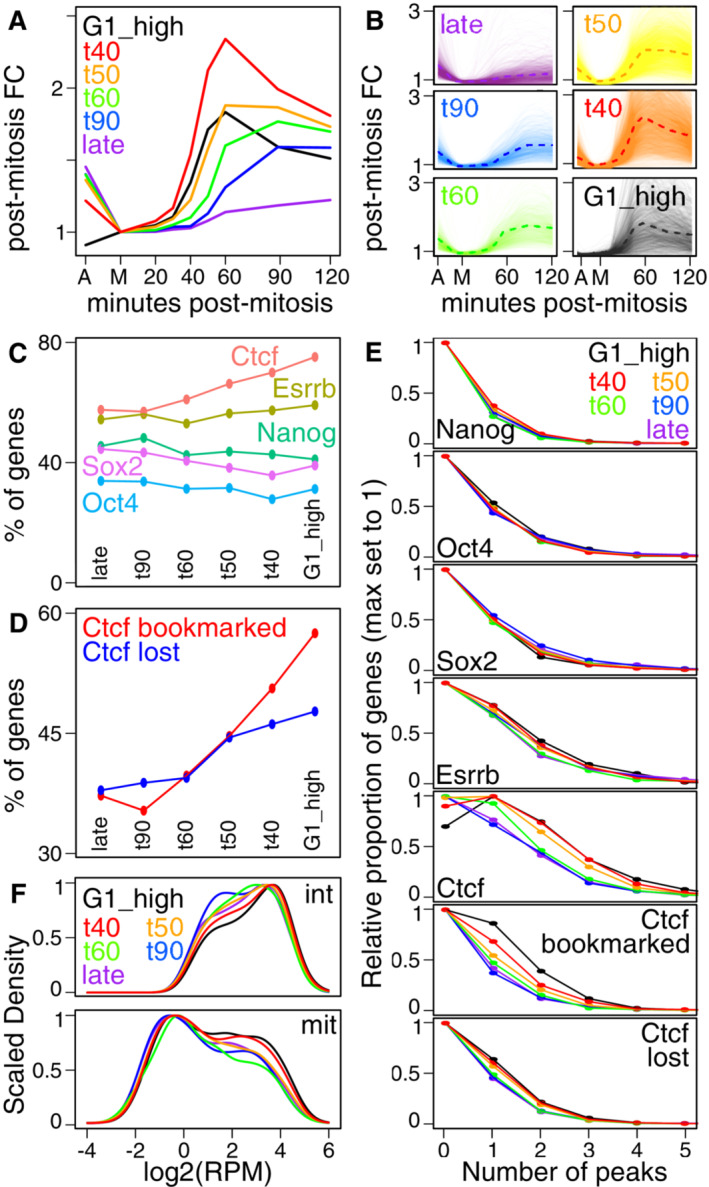
CTCF binding in interphase and mitosis correlates with fast post‐mitotic gene reactivation Average post‐mitotic fold change of gene groups identified by the time at which the levels detected in asynchronous cells are reached or surpassed, as shown in Fig 3B (G1_high: 831 genes with low detected levels in asynchronous cells; t40: 3,188 reaching or surpassing asynchronous levels by 40 min post‐mitosis; same for t50–t90: 3,362, 647, 762; late: genes reaching asynchronous levels after 120 min (88) or never within the analysed post‐mitotic timepoints (1,033).Individual gene traces (light lines; smoothed with a loess regression) corresponding to each category presented in (A), and respective averages (dashed lines).Percentage of genes presenting at least 1 TF binding site as detected by ChIP‐seq within 25 kb centred on the TSS, calculated for each gene category shown in (A).Similar analysis to (C) but computing the percentage of genes with mitotically bookmarked and lost CTCF binding sites. Genes were counted as bookmarked or lost when at least one or no CTCF binding site was detected in interphase and mitosis, respectively.The relative proportion of genes for each reactivation category shown in (A) as a function of the number of binding sites (*X*‐axis) for the indicated TFs. Binding sites overlapping a 25 kb region centred on the TSS were considered. All distributions are scaled to the maximal proportion, observed for zero sites for all TFs, except for CTCF, for which it was at one site for rapidly reactivating gene groups.Distribution of CTCF binding levels in interphase (top) and in mitosis (bottom) for each gene group shown in (A). For each gene, all the binding sites overlapping a 25 kb region centred on the TSS were considered. Binding levels (*X*‐axis) are expressed as reads per million (RPM).

We next aimed at exploring whether gene reactivation after mitosis was correlated with the binding of specific TFs, which are the main drivers of ES cell self‐renewal and pluripotency. To do this, we selected four groups of genes reaching or surpassing the levels detected in asynchronous cells (as in Fig 3B, right panel) at 40 (*n* = 3,188 genes), 50 (*n* = 3,362), 60 (*n* = 647) or 90 (*n* = 762) minutes post‐mitosis, a fifth group (labelled late in Fig 4A and B; *n* = 1,121) that never reached the levels detected in asynchronous cells and a sixth group showing low levels in asynchronous cells but a marked increase after mitosis (labelled G1_high in Fig 4A and B; *n* = 831). Next, we computed the percentage of genes of each group bound by pluripotency TFs – Nanog, Oct4, Sox2 and Esrrb (Festuccia *et al*, 2019; Heurtier *et al*, 2019) – or by the architectural TF CTCF (Merkenschlager & Nora, 2016; Owens *et al*, 2019) within a 25 kb region centred on the transcription start site. Strikingly, only CTCF clearly showed increased binding near genes reactivating fast (Fig 4C; other genomic sizes than 25 kb gave similar results, Appendix Fig S4A). Since CTCF has been shown to display robust mitotic binding at a large subset of its interphase targets in ES cells (Owens *et al*, 2019), we further distinguished between genes associated with CTCF during mitosis or only during interphase (bookmarked and lost, respectively, in Fig 4D). We found a higher enrichment of bookmarked compared with lost sites for both the most rapidly reactivated (t40) and the G1_high groups. Moreover, when we counted the number of distinct TF binding sites within 25 kb centred on each promoter (Fig 4E), we found a similar distribution among reactivation groups for all TFs except for CTCF. An increased proportion of genes in the fast‐reactivating groups (t40, t50 and G1_high groups) associated with three or more binding sites, an effect that is attributable specifically to bookmarked, and not lost, CTCF sites (Fig 4E). Finally, we computed the distribution of CTCF binding levels in interphase and mitosis in proximity of each group of genes (Fig 4F) and observed that fast reactivating groups (t40 and G1_high) are partially depleted of regions showing low levels of CTCF binding. Regions characterised by high binding levels in mitosis are instead enriched near these genes. Taken together, these correlative analyses point to CTCF as a regulator of the reactivation dynamics after mitosis. Notably, the potential effects of CTCF binding are particularly prominent for G1_high genes, indicating a specific function after mitosis for this TF, which can be uncoupled from its activity during interphase.

### Robust post‐mitotic transcriptional reactivation in the absence of CTCF

**Figure 5:**
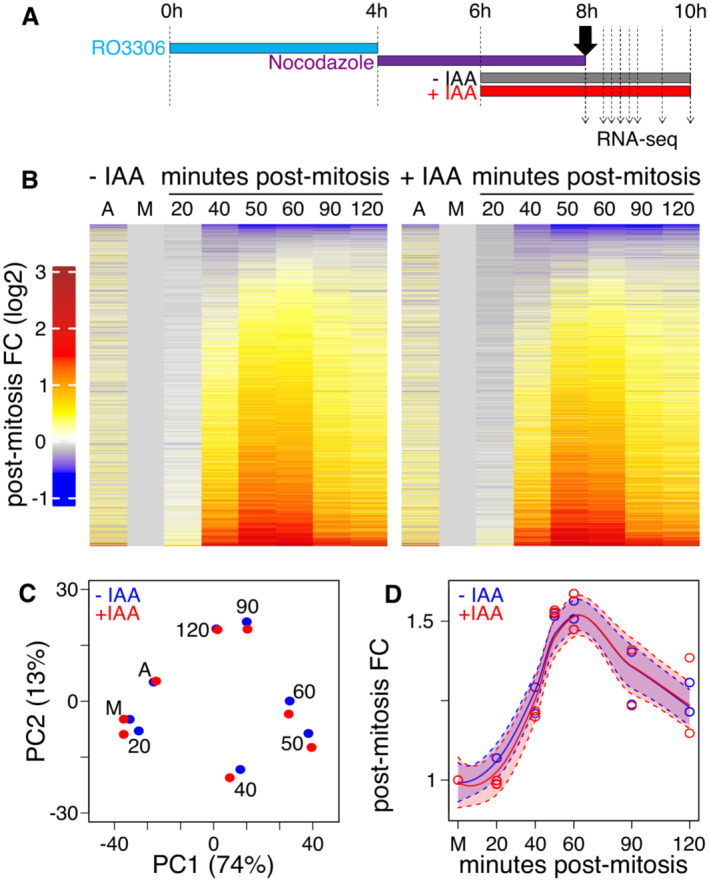
CTCF does not influence global post‐mitotic gene reactivation Schematic describing the experimental layout for IAA‐induced depletion of CTCF at the M/G1 transition.Heatmaps presented like in Fig 3A for untreated (−IAA; left) or CTCF‐depleted (+IAA; right) CTCF‐AID cells.PCA of post‐mitotic gene reactivation in the presence (−IAA; blue) or absence of CTCF (+IAA; red).Smoothed (loess) median reactivation kinetics in the presence (−IAA; blue) or absence of CTCF (+IAA; red). The shadow areas represent 95% confidence intervals of the regression.

To test the role of CTCF in post‐mitotic gene reactivation, we took advantage of CTCF‐AID ES cells (Nora *et al*, 2017), which enable fast (~2 h) and acute degradation of CTCF upon auxin (IAA) treatment (Owens *et al*, 2019). After verifying that ES cells synchronised in mitosis with the double inhibition protocol mantain robust mitotic CTCF binding at the regions previously identified (Owens *et al*, 2019; Appendix Fig S4B), we applied it to CTCF‐AID ES cells, which were either treated or not with IAA during the last 2 h of nocodazole treatment to deplete CTCF. Subsequently, mitotic CTCF‐AID cells were shaked‐off and re‐seeded in nocodazole‐free medium −/+ IAA such that they completed mitosis and re‐entered interphase with or without CTCF (Fig 5A and Appendix Fig S4C). Despite the global association of gene reactivation with CTCF binding (Fig 4), very similar profiles were observed in the presence (−IAA) or absence (+IAA) of CTCF (Fig 5B and Dataset EV3). Principal Component Analysis (PCA) revealed similar trajectories of the +/−IAA transcriptomes (Fig 5C), leading to nearly identical median gene reactivation dynamics (Fig 5D). These results indicate that the post‐mitotic burst in ES cells is robust and largely insensitive to CTCF depletion, as recently shown in other biological contexts (Thiecke *et al*, 2020; Zhang *et al*, 2021a). Nevertheless, careful examination of the PCA (Fig 5C) suggests that small differences exist along PC1 between + and −IAA treatments, 20 and 40 min after mitosis. At later time‐points, small differences appear instead in PC2. In contrast, both +/−IAA datasets in asynchronous and 2 h post‐mitosis showed identical PC1 and PC2 scores, providing an internal control to the small but measurable effects mediated by CTCF. Hence, while the global effect of CTCF following mitosis is undeniably small, these observations prompted us to identify gene‐specific responses that could be more directly associated with CTCF binding, especially in mitotic cells.

### Mitotic bookmarking of CTCF is required for proper reactivation of selected promoter‐proximal target genes

**Figure 6:**
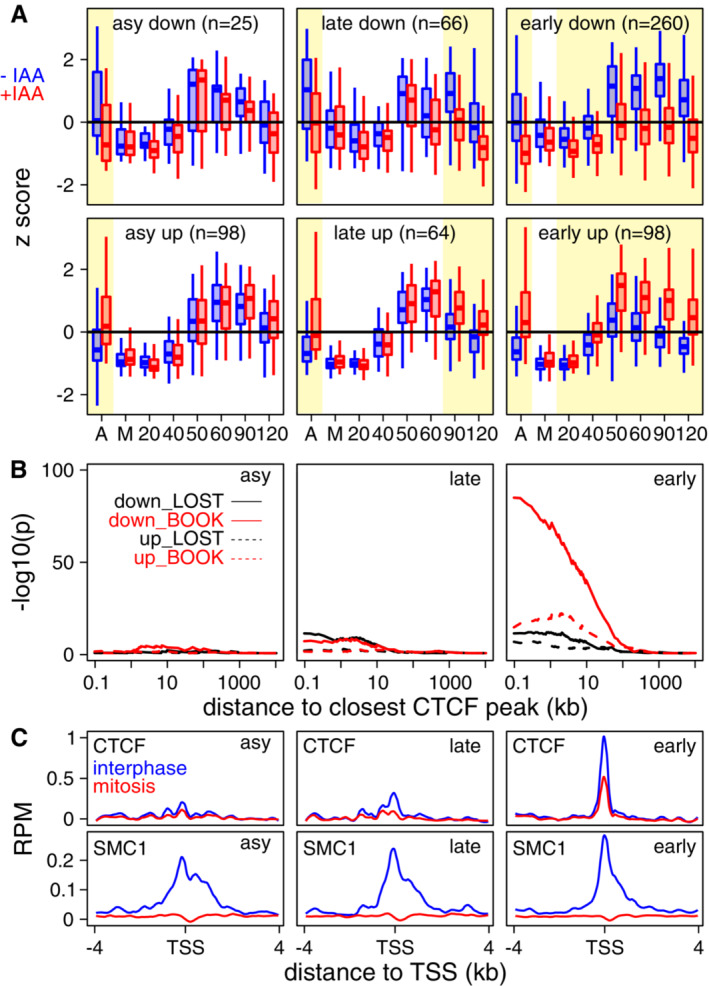
Mitotic bookmarking effects mediated by CTCF Gene expression changes (*z*‐score) in asynchronous cells and during post‐mitotic release of CTCF‐AID cells treated or not with IAA to deplete CTCF (blue, presence of CTCF; red, absence). Samples are indicated on the *X*‐axis (A: asynchronous cells; M: mitotic cells; numbers: minutes since release from mitotic block). Six gene groups are shown, based on the time of their response to CTCF depletion (asy, late, early) and the direction of the transcriptional change (up, down). The number of genes of each group is indicated. The yellow shadow areas indicate times showing expression changes. The boxplots show the median (bar), 25–75% percentiles (box) and 1.5‐fold inter‐quartile range (whiskers).Statistical enrichment (*Y*‐axis, −log10(Fisher *P*‐value)) of CTCF binding sites at increasing distances (log10(kb)) of the TSS of genes responding to CTCF depletion exclusively in asynchronous cells (asy; left) or either late (middle) or early (right) after mitotic release. Two categories of CTCF sites are separately analysed: those that are present in interphase and mitosis (Book, in red) and those that are exclusive of interphase (Lost, in black). The direction of gene responses is also considered (down and up, see (A), as solid and dashed lines respectively).Average binding profile (*X*‐axis, reads per million) of CTCF (top) and the Cohesin subunit SMC1 (bottom), across 8 kb centred on the TSS of genes responding to CTCF depletion exclusively in asynchronous cells (left) or either late or early upon mitotic release (middle and right, respectively). Interphase is shown in blue and mitosis in red.

Using standard RNA‐seq differential expression analysis, we identified 611 genes displaying differential pre‐mRNA expression in +/−IAA in either asynchronous or in at least two post‐mitosis samples (Fig 6A and Dataset EV3). These 611 genes were further divided into genes being up‐ or downregulated upon the loss of CTCF and by their temporal response: exclusively in asynchronous cells, or also at either late or early time‐points post‐mitosis (asy, late and early in Fig 6A, with examples in Appendix Fig S5A). Among all six categories, genes activated by CTCF from early post‐mitotic time‐points (early_down) were prominent (260 genes). Gene ontology analyses, however, did not identify any relevant term being enriched for any of the identified groups (Appendix Fig S5B). We conclude, therefore, that while at a global scale, the loss of CTCF in mitosis and early interphase is largely inconsequential, a sizeable fraction of genes clearly depends on CTCF to be efficiently reactivated. Next, we explored whether the different speeds of response to CTCF after mitosis were associated to CTCF binding. For this, we calculated the percentage of genes bound by CTCF and found that around 50% of early responders were associated with promoter‐proximal binding in mitosis (Appendix Fig S6A). To further expand this analysis, we computed Fisher exact tests for the enrichment of each group of differentially expressed genes at increasing distances to the nearest bookmarked or mitotically lost CTCF peak (Fig 6B). While asy genes were not enriched, suggestive of indirect effects, we observed that genes activated by CTCF late after mitosis (late‐down) displayed low enrichment levels, including near Lost CTCF binding sites. Hence, at these genes, the reacquisition of CTCF binding after mitosis is accompanied by their sustained activation, indicating that even in the absence of mitotic binding, CTCF may contribute to gene activity before the onset of replication. This interpretation is supported by the fast rebinding of CTCF to DNA following mitosis (Zhang *et al*, 2019, 2021a). Strikingly, genes responding rapidly after mitosis to the loss of CTCF displayed a strong statistical association to CTCF binding sites, with a very prominent enrichment of genes activated by CTCF (early‐down) in the vicinity of Book sites (Fig 6B). This indicates that promoter‐proximal bookmarked CTCF sites lead to CTCF‐dependent transcription inmediately after division for this subset of responsive genes. Thus, mitotic bookmarking confers almost immediate post‐mitotic responsiveness to CTCF compared with other non‐bookmarked but CTCF‐dependent genes. To further confirm these statistical correlations, we directly assessed CTCF binding in interphase and in mitosis at asy‐only, late and fast responders, using available ChIP‐seq data (Owens *et al*, 2019). Robust binding at the TSS in both interphase and mitosis was observed at early‐responders exclusively, whereas Cohesin (Smc1; Merkenschlager & Nora, 2016) was similarly associated with the three groups (Fig 6C). These data strongly suggest that promoter‐proximal CTCF binding in mitosis is associated with early activatory function, demonstrating the mitotic bookmarking role of CTCF in mouse ES cells. Moreover, a lower but significant enrichment of CTCF Book sites was also observed for genes being rapidly repressed by CTCF (early‐up; Fig 6B) indicating that mitotic CTCF binding may also be used as a mean for bookmarking for repression.

## Discussion

Here, we aimed at characterising how ES cells reactivate their genome after mitosis. While several studies have already addressed this question (Festuccia *et al*, 2016, 2019; Teves *et al*, 2018; Pelham‐Webb *et al*, 2021), none had the temporal resolution we achieved. We observe that the vast majority of active genes undergoes a prominent transcriptional burst largelly surpassing the levels detected in asynchronous cells. A post‐mitotic hyper‐active state has now been observed in different cell types (Hsiung *et al*, 2016; Palozola *et al*, 2017; Kang *et al*, 2020), suggesting that it may represent a general feature. The drivers of this phenomenon remain to be directly identified. Two scenarios can be envisioned. First, the burst observed in G1 could depend on specific and direct mechanisms, such as the behaviour of general and/or ubiquitous TFs executing such function. For instance, the sudden relief of inhibitory phosphorylations on many components of the basal transcriptional machinery might contribute to a synchronous activation of transcription (Segil *et al*, 1996; Akoulitchev & Reinberg, 1998). Moreover, sequence‐specific TFs involved in hyper‐transcription in ES cells, but possibly also in other cell types, such as *c‐Myc* (Efroni *et al*, 2008; Percharde *et al*, 2017), could also represent important candidates (Appendix Fig S7). Second, the post‐mitotic hyper‐active transcriptional state could be the indirect consequence of other properties of post‐mitotic cells. For instance, the re‐folding of the genome into topologically associating domains (TADs), which largely confine enhancer function, has been shown to occurr, in average, after gene reactivation (Zhang *et al*, 2019, 2021a, 2021b). Importantly, before TADs are re‐established, intra and inter‐TAD enhancer‐promoter contacts drive inappropriate gene activation (Zhang *et al*, 2019). Hence, generally increased enhancer‐promoter interactions, perhaps transiently and randomly established during the transition from a Condensin‐mediated mode of 3D organisation in metaphase, to one driven by Cohesin in telophase (Abramo *et al*, 2019), could fuel the burst of transcription observed in G1. However, we show that a main driver of TADs formation, CTCF, does not influence the global dynamics of reactivation, neither in ES cells nor in somatic cells. Additional properties, such as a sudden decondensation of the chromatin, should be envisioned as potential drivers. Understanding whether post‐mitotic transcriptional hyper‐activity is the primary result of specific regulations and/or a secondary effect of other cellular changes will require additional studies.

Compared with published studies performed in other cell types (Hsiung *et al*, 2016; Palozola *et al*, 2017; Kang *et al*, 2020), ES cells appear to reactivate their genome faster as they exit from mitosis. CTCF fulfils key characteristics as a suitable candidate to explain this particularly expedite reactivation: it displays extensive mitotic binding specifically in ES cells (Owens *et al*, 2019) and is frequently bound in the vicinity of rapidly reactivated genes. Despite these correlations, our data show that the post‐mitotic burst of transcription occurs in ES cells with overall similar dynamics in the presence or absence of this factor. Therefore, other mechanisms of post‐mitotic acceleration should be investigated. Moreover, our analyses have also shown that the most strongly induced genes after mitosis correspond to genes associated with the G1/S transition and DNA replication, an observation that is in line with the lack of a G1/S checkpoint in ES cells. Indeed, establishing a functional replication machinery rapidly after mitosis seems a major requirement to efficiently and productively enter into the S phase. Despite this, the temporal differences with other functional categories are minor, below 20 min. Therefore, ES cells seem to simultaneously reactivate cell identity and ubiquitous genes, in contrast with other cell types (Hsiung *et al*, 2016; Palozola *et al*, 2017; Kang *et al*, 2020). This faster and indistinctly global reactivation in post‐mitotic ES cells may have evolved as a response to two requirements: one, undergo fast proliferation, achieved by the lack of a G1/S checkpoint; two, preserve a continuosly active regulatory network – even subtle changes in TF dosage have been shown to induce the loss of self‐renewal and to promote differentiation. Hence, it seems reasonable to think that to execute multiple tasks efficiently before entering replication, ES cells may accelerate the global rate of gene reactivation compared to somatic cells that can rely on decision‐making mechanisms to transit to the S phase. Nevertheless, caution must be taken as differences between the methods used to synchronise cells or to measure transcriptional outputs may bias, at least partially, the conclusion drawn by direct comparison between studies. However, the fact that ES cells reactivate faster than other cell types is in line with their general hyper‐transcription (Efroni *et al*, 2008) and permissive chromatin state (Meshorer *et al*, 2006). Whether these properties are alone sufficient to accelerate post‐mitotic gene transcription requires further investigation.

Despite not having global effects, CTCF exerts, however, a clear influence on around 350 genes where it binds at or nearby the promoter, both in interphase and in mitosis. For more than 250 of these genes, mitotic bookmarking by CTCF is required for proper reactivation as soon as 20 min post‐release; for around 100, it is conversely required to attenuate transcription early in G1. While it remains unclear how mitotic CTCF binding conveys either activatory or repressive function, these observations experimentally establish that CTCF acts as a mitotic bookmarking factor, as previously suggested (Owens *et al*, 2019). Hence, while its main function is generally considered that of shaping the global architecture of the genome, this study underscores the function of CTCF as a gene‐specific regulator displaying a key role in the control of selected genes as cells exit mitosis. Whether other mitotic bookmarking factors perform similar functions remains to be directly assessed. Moreover, this study calls for reconsidering, at least partially, the role of mitotic bookmarking TFs, long considered a potential mechanism to accelerate gene reactivation after division. However, the fast and efficient reactivation of the whole transcriptome – particularly in ES cells but also in other cell types – raises the question of the real advantage conferred by this phenomenon. In other words, does anticipating the resumption of gene expression by few minutes, and for a relatively small subset of genes, play a determinant role in preserving cell function? Perhaps counterintuitively, our data argue against the simple idea of mitotic bookmarking as an accelerator of gene reactivation. Indeed, while rapidly reactivated genes are in average more frequently associated with mitotic CTCF binding, comparing the reactivation dynamics of genes bookmarked by CTCF to those bound by CTCF exclusively in interphase, we observe similar reactivation profiles (Appendix Fig S6B). We observe instead that bookmarked genes are responsive to CTCF early after mitosis, while non‐bookmarked genes are much less prone to CTCF‐dependent regulation, even if both sets respond to CTCF in asynchronous cells. Therefore, the main effect of mitotic bookmarking does not seem to be changing the timing but, rather, the amplitude of gene reactivation. Whether this is a specificity of CTCF or a general and unapreciated intrinsic feature of mitotic bookmarking by TFs will be an important question for future studies.

## Materials and Methods

### Cell culture and mitotic preparations

ES cells – CCNA‐GFP (Festuccia *et al*, 2016) and CTCF‐AID #EN52.9.1 (Nora *et al*, 2017), both derived from mouse E14Tg2a strain 129/Ola – were cultured on standard serum and LIF conditions as previously described (Owens *et al*, 2019), passaged 1:10 every 2–3 days and regularly tested mycoplasma‐free. To obtain mitotic ES cells (> 95% purity as assessed by DAPI staining and microscopy), we used a double synchronisation method. Briefly, cells were cultured for 4 h in the presence of 10 μM of the CDK1 inhibitor RO‐3306 (Sigma, SML0569), washed in PBS and incubated in medium supplemented with 50 ng/ml nocodazole (Sigma, M1404), for an additional 4 h. Cells were briefly washed in PBS, and a gentle shake‐off was then performed in 10 ml of PBS, monitoring the process under a microscope to avoid detaching clumps of cells in interphase. The cell suspension was filtered through a 10 μm filter (pluriSelect, 43‐50,010‐03) by gravity and cells spun down, resuspended in pre‐warmed medium and immediately transferred to the incubator in pre‐warmed 3 cm dishes, which were intentionally not coated with gelatine. For each timepoint/treatment, 1–2 × 10^6^ cells were seeded in separate dishes in a volume of 1 ml, so that upon collection only the cells to be harvested were taken out of the incubator. Cells were detached from the dish by pipetting, rapidly spun down at 4°C (1 min, 150 *g* in a benchtop centrifuge) and lysed in 500 μl of cold TRIzol (ThermoFisher, 15596026). The procedure was also monitored by FACS analysis of CCNA‐GFP, cultured in the presence of 20 μM Hoechst‐33342 for 20 min, trypsinised, filtered through a 40 μm cell strainer and kept on ice. Hoechst and GFP levels were analysed using a LSR Fortessa instrument (Becton–Dickinson) and the FlowJo software suite (Tree Star). The timing of initiation of DNA replication was determined in cells released from a double mitotic synchronisation into EdU‐containing medium (10 μM; ThermoFisher, A10044). EdU was revealed after formaldehyde fixation (4%), permeabilisation (PBS1× 0.1% Triton X‐100) and staining (30 min with 100 mM Tris, 2 mM CuSO_4_, 2.5 μM Alexa‐fluor 488 azide (ThermoFisher, A10266), 100 mM ascorbic acid). CTCF depletion in CTCF‐AID cells was achieved with 0.5 mM auxin (IAA Sigma, I5148) for 2 h, which we showed previously leads to an acute depletion (Owens *et al*, 2019), starting after 2 h of nocodazole treatment. IAA was maintained throughout the whole release procedure. Asynchronous cells were treated in parallel during 4 h. For each cell line and condition, we generated three independent replicates at the indicated times. All samples of one replicate were sequenced; for the other two, asynchronous and mitotic cells were sequenced together with alternating timepoints during the release (Datasets EV1 and EV3).

### RNA‐seq

RNA was extracted with 500 μl TRIzol (ThermoFisher, 15596026), according to the manufacturer's protocol, and treated with DNAse I (Qiagen) for 20 min at 37°C. Following phenol:chloroform purification, RNAs were resuspended in Ultrapure DNAse/RNase Free Distilled Water (Thermo, 10977035). Ribo‐depleted, stranded and paired‐end RNA‐seq libraries were prepared and sequenced by Novogene Co Ltd. Reads were aligned to the mm10 genome using STAR (Dobin *et al*, 2013), quantified by RSEM (Li & Dewey, 2011) and counts transformed into Transcripts per Million (TPM, Datasets EV1 and EV3). Gencode (vM12) gtf was used after adding a pre‐mRNA isoform for each annotated gene, spanning the longest isoform of all available mRNA isoforms.

### ChIP‐seq

CTCF ChIP‐seq was performed as previously described (Owens *et al*, 2019) but using chromatin prepared from cells arrested in mitosis using the double CDK1‐Nocodazole protocole described above. Briefly, formaldehyde cross‐linked chromatin (1%, 10 min) was sonicated with a Bioruptor Pico (Diagenode) and immunoprecipitated with anti‐CTCF antibodies (Active Motif 61311). Precipitated DNA was used for library prepartions (Festuccia *et al*, 2019) and sequenced in the Biomics facility of the Institut Pasteur.

### Bioinformatic analyses

Analyses were performed in R (version 3.6.3). We considered genes displaying at least a mean TPM > 0.5 in either asynchronous or in at least two samples post‐mitosis and present in the Refseq curated repository downloaded from the UCSC. Post‐mitotic fold‐changes were calculated per replicate and then averaged to generate gene expression plots and heatmaps using ComplexHeatmap package (Gu *et al*, 2016). Smoothed gene expression profiles, used for visualisation purposes only, were obtained with a loess regression. Principal component analysis was run with prcomp function with centred data corresponding to post‐mitotic log2(FC) for CCNA‐GFP and log2(TPM) for CTCF‐AID to capture direct differences between IAA treatments. Gene ontology and gene set enrichment analyses were performed using the enrichR package (Xie *et al*, 2021). To correlate genes and TF binding events and to compute CTCF and SMC1 binding profiles, we first selected a single TSS for each gene, which corresponded to the TSS displaying highest levels of RNAPII, H3K4me3 and DNaseI accessibility using available ES cell data from the Encode consortium. TF binding sites were all previously reported (Festuccia *et al*, 2019; Heurtier *et al*, 2019; Owens *et al*, 2019). Fisher‐exact tests to assess gene‐TF proximity enrichments were performed as previously described (Heurtier *et al*, 2019). ChIP‐seq enrichments were calculated using the bamsignals package. Differentially expressed genes were identified using rounded RSEM counts per sample and DESeq2 (Love *et al*, 2014). Pre‐mRNAs with FDR < 0.1 and absolute log2 Fold‐Change > 0.2 in either asynchronous cells or in at least two post‐mitotic samples were considered as differentially expressed.

## Author contributions

**Almira Chervova:** Data curation; formal analysis; investigation. **Nicola Festuccia:** Investigation; methodology; writing – review and editing. **Luis Altamirano‐Pacheco:** Data curation; formal analysis. **Agnès Dubois:** Resources. **Pablo Navarro:** Conceptualization; formal analysis; supervision; funding acquisition; visualization; writing – original draft; project administration.

## Disclosure and competing interests statement

The authors declare that they have no conflict of interest.

## Supporting information



Appendix

Dataset EV1

Dataset EV2

Dataset EV3

## Data Availability

The RNA‐seq dataset produced in this study is available in the following database:
RNA‐Seq data: Gene Expression Omnibus GSE196889 (https://www.ncbi.nlm.nih.gov/geo/query/acc.cgi?acc=GSE196889).
