# Supplementary material for: A gene subset requires CTCF bookmarking during the fast post‐mitotic reactivation of mouse ES cells
Source: EMBO Rep. 2022 Nov 4;24(1):e56075. doi: 10.15252/embr.202256075 (PMC9827546; doi:10.15252/embr.202256075)
Supplement: Supplementary file 1 — Appendix [file EMBR-24-e56075-s001.pdf]

Appendix

Chervova A and Festuccia N et al.

Fast post-mitotic gene reactivation of ES cells
and the role of the mitotic bookmarking factor CTCF

Table of Content:

Appendix Figure S1	Page 2
Appendix Figure S2	Page 3
Appendix Figure S3	Page 4
Appendix Figure S4	Page 5
Appendix Figure S5	Page 6
Appendix Figure S6	Page 7
Appendix Figure S7	Page 8

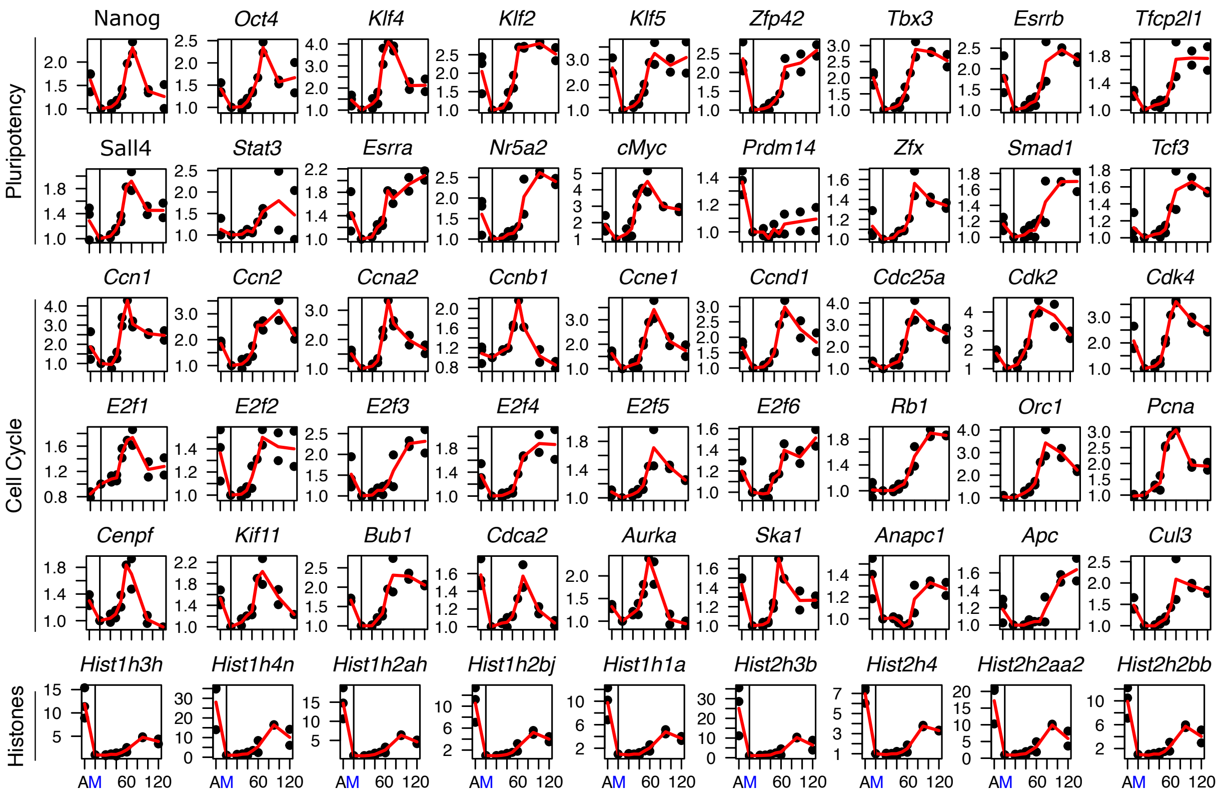

Figure S1. Examples of transcriptional profiles for individual genes. For each plot, each dot represents a replicate and the red line the corresponding average. Y-axis shows fold change to mitosis; X-axis the samples (A: asynchronous cells; M: mitotic cells; numbers: minutes after release). For all genes except for histones, pre-mRNA levels are shown.

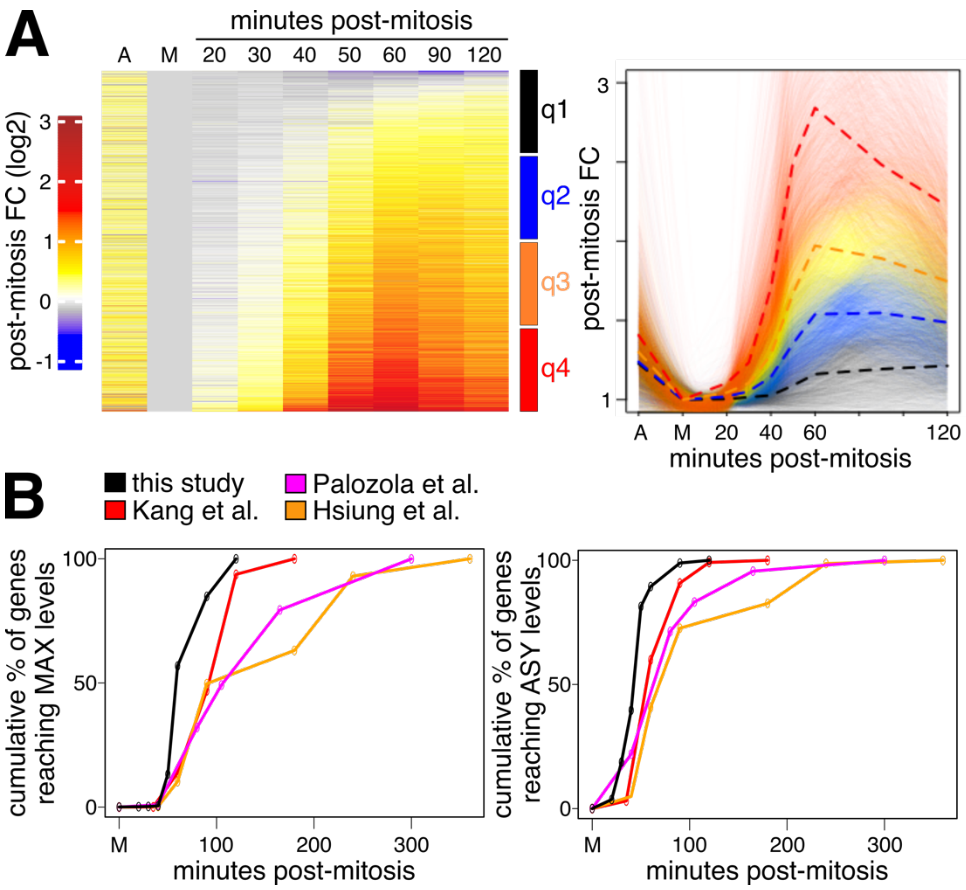

Figure S2. Fast post-mitotic reactivation in ES cells. (A) The heatmap on the left is identical to that described in Figure 3A, shown here to illustrate the quantiles (q1, black; q2, blue; q3, orange; q4, red) used on the right. The plot shows the individual traces and the mean of all genes belonging to q1, q2, q3 and q4, illustrating that the heatmap orders genes both by the timing and the amplitude of post-mitotic transcription. **(B)** Comparison of gene reactivation dynamics in ES cells (this study, black) with previous work (*Hsiung et al*, 2016; *Palozola et al*, 2017; *Kang et al*, 2020), presented as in Figure 3B.

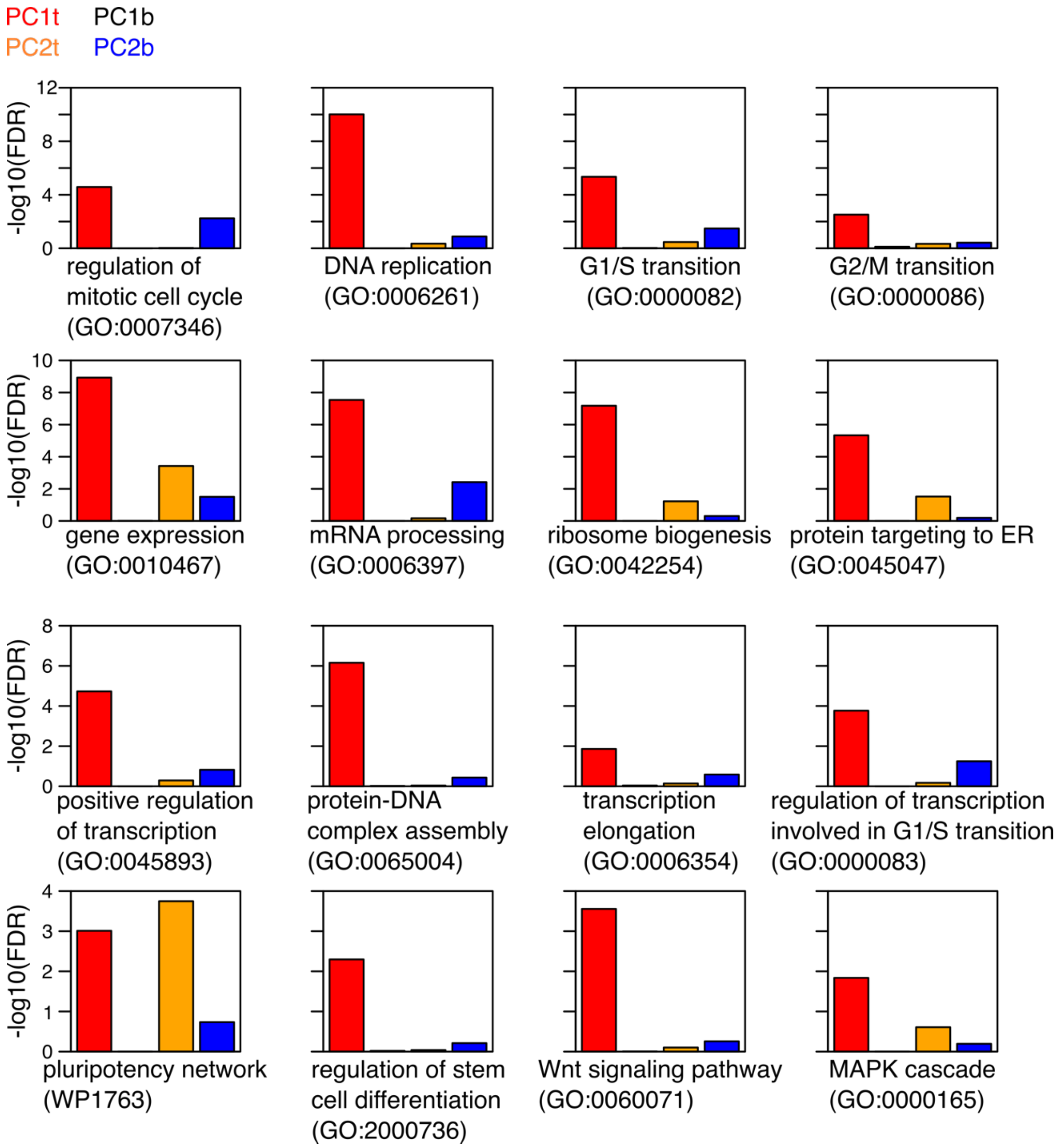

Figure S3. Examples of gene ontology associations on classes of genes showing different post-mitotic behaviour. Gene ontology enrichments (-log10(FDR)) for cell-cycle categories (first row), gene regulation (second and third rows) and stem cell related terms (forth row), calculated for 4 lists of genes derived from PCA analysis of post-mitotic gene reactivation datasets. PC1t, PC1b, PC2t, PC2b correspond to the gene groups shown in Figure 3.

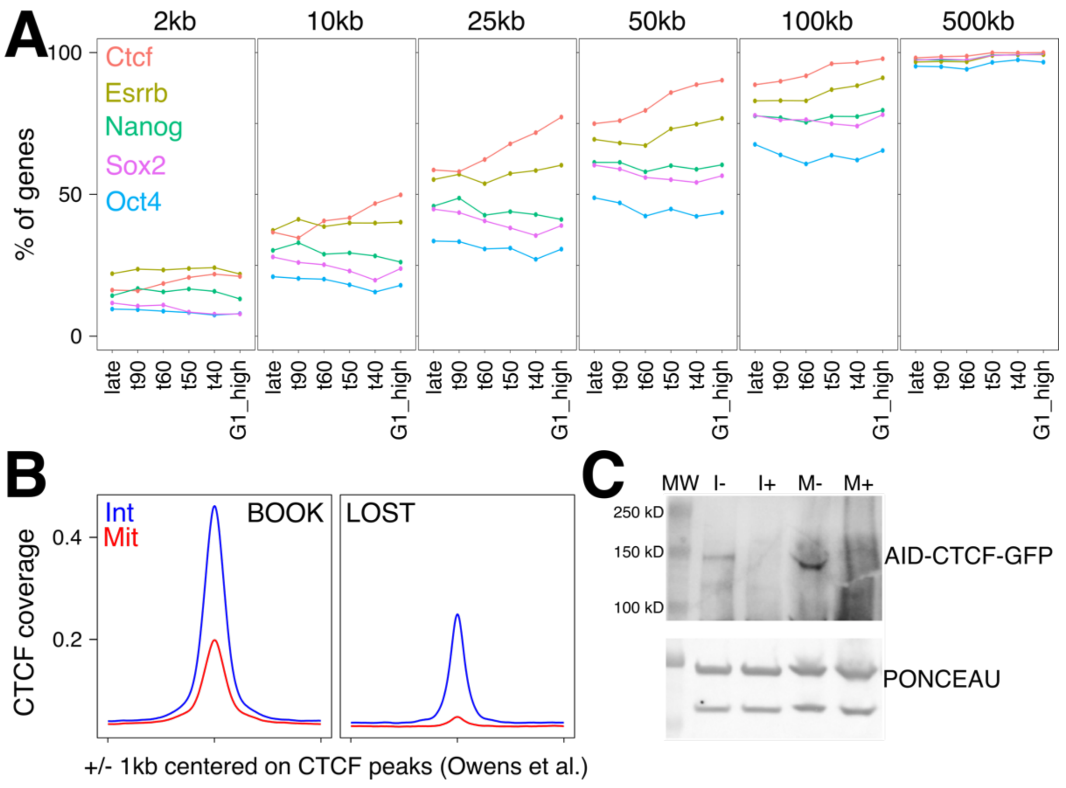

Figure S4. Additional analyses of pluripotency TFs and CTCF. (A) Percentage of genes presenting at least 1 TF binding site as detected by ChIP-seq within increasingly bigger genomic windows centred on the TSS, as indicated, calculated for each gene category as in Figure 4C. **(B)** ChIP-seq average binding profile of CTCF at previously identified bookmarked/lost regions (*Owens et al*, 2019), using a double inhibition protocol (RO3306 and Nocodazole) to obtain mitotic cells (red), compared to asynchronous cells (blue). **(C)** Western Blot showing CTCF depletion upon auxin treatment in interphase (I) and mitosis (M).

A

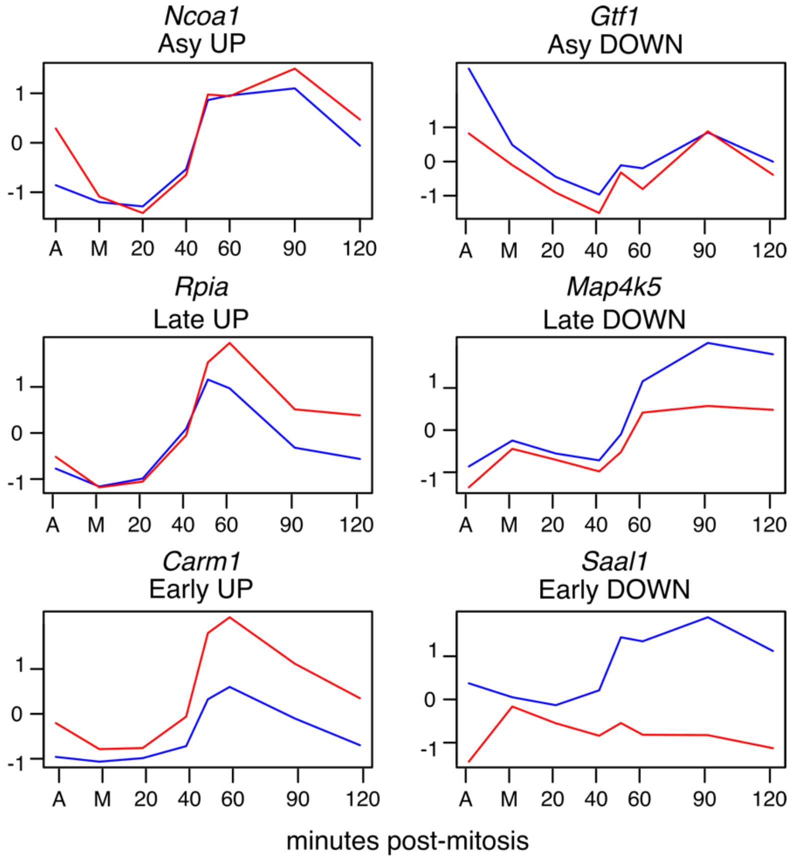

B

Term (Biol. Proc. - Mol. Func. - Cel. Comp.)	Gene Group	Overlap	FDR
RNA binding (GO:0003723)	down	47/1406	0.00605745156311219
aryl hydrocarbon receptor binding (GO:0017162)	up	3/8	0.0376630558933324
sodium:bicarbonate symporter activity (GO:0008510)	slow	2/6	0.0260840345590211
solute:bicarbonate symporter activity (GO:0140410)	slow	2/6	0.0260840345590211
transmembrane receptor protein kinase activity (GO:0019199)	slow	4/60	0.0260840345590211
transmembrane receptor protein tyrosine kinase activity (GO:0004714)	slow	4/60	0.0260840345590211
protein tyrosine kinase activity (GO:0004713)	slow	5/108	0.0260840345590211
sodium:bicarbonate symporter activity (GO:0008510)	down_slow	2/6	0.0098098056717529
solute:bicarbonate symporter activity (GO:0140410)	down_slow	2/6	0.0098098056717529
ribonucleoprotein complex assembly (GO:0022618)	down_fast	10/136	0.0209500257459238
RNA binding (GO:0003723)	down_fast	36/1406	0.0235126268993246
positive regulation of protein acetylation (GO:1901985)	up_asy	3/13	0.0330274738295088
protein localization to cell junction (GO:1902414)	up_asy	3/22	0.0424179264636465
monocarboxylic acid transport (GO:0015718)	up_asy	4/57	0.0424179264636465
negative regulation of gene expression (GO:0010629)	up_asy	8/322	0.0424179264636465
positive regulation of fibroblast growth factor receptor signaling pathway (GO:0045743)	up_asy	2/5	0.0424179264636465
peripheral nervous system development (GO:0007422)	up_asy	3/25	0.0424179264636465
aryl hydrocarbon receptor binding (GO:0017162)	up_asy	3/8	0.0013115982352832
nuclear receptor coactivator activity (GO:0030374)	up_asy	4/53	0.0137961063559994
intracellular membrane-bounded organelle (GO:0043231)	up_asy	45/5192	0.00169231556322617
nucleus (GO:0005634)	up_asy	39/4484	0.00444343156861381
transmembrane receptor protein kinase activity (GO:0019199)	up_slow	3/60	0.0360631636263514
transmembrane receptor protein tyrosine kinase activity (GO:0004714)	up_slow	3/60	0.0360631636263514
1-phosphatidylinositol binding (GO:0005545)	up_slow	2/15	0.0360631636263514

Figure S5. Examples and functional gene groups of pre-mRNAs responding to CTCF depletion. (A) Examples of individual genes belonging to the CTCF responsive gene groups identified in Figure 6A. **(B)** Table of all gene ontology terms (FDR<0.05) identified when considering different combinations of genes derived from Figure 6A, as indicated (Gene Group).

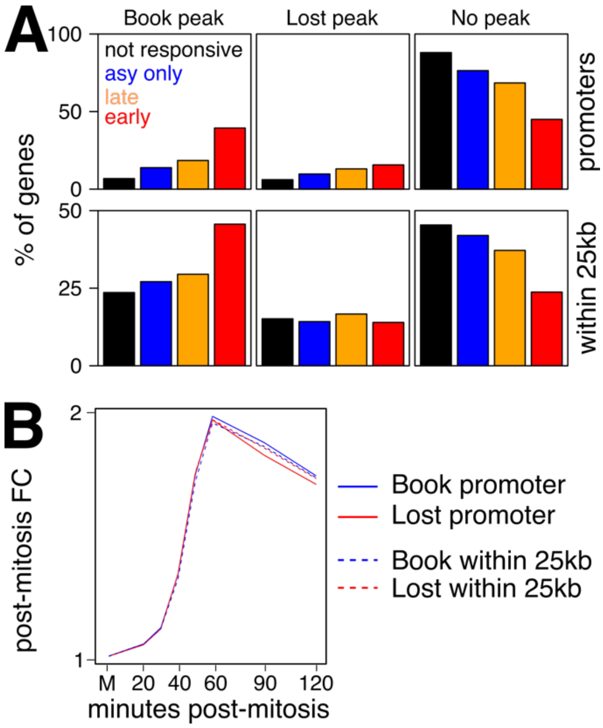

Figure S6. Additional correlations of CTCF bookmarking with post-mitotic gene transcription. (A) Percentage of genes displaying CTCF binding in interphase and in mitosis (Book, left panel), in interphase only (Lost, middle panel), or none (right panel), as a function of their dependency to CTCF: not responsive (black), responsive only in asynchronous cells (blue) or also early (red) or late (orange) after mitosis. Two parameters were considered: binding at the promoter (top) and within a 25kb region spanning the promoter (bottom). **(B)** Average post-mitotic dynamics of genes bound by CTCF either in interphase and in mitosis (Book, red) or only in interphase (Lost, blue). Two parameters were considered: binding at the promoter (solid lines) and within a 25kb region spanning the promoter (dashed lines).

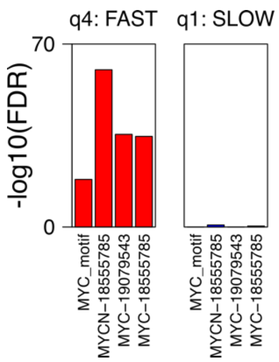

Figure S7. MYC is a relevant candidate to control post-mitotic gene transcription in ES cells. Statistical enrichments of two groups of post-mitotic reactivation genes (fast and slow, corresponding to q4 and q1 in Figure S2 A). The analysed parameters are the presence of the MYC motif at the TSS, or the presence of MYC ChIP-seq peaks in PMID-18555785, PMID-19079543, PMID-18555785, as reported by Enrichr analyses.
