# Supplementary material for: A gene subset requires CTCF bookmarking during the fast post‐mitotic reactivation of mouse ES cells
Source: EMBO Rep. 2022 Nov 4;24(1):e56075. doi: 10.15252/embr.202256075 (PMC9827546; doi:10.15252/embr.202256075)
Supplement: Supplementary file 5 [file EMBR-24-e56075-s003.pdf]

A gene subset requires CTCF bookmarking during the fast post mitotic reactivation of mouse ES cells

Almira Chervova, Nicola Festuccia, Luis Altamirano-Pacheco, Agnès Dubois, and Pablo Navarro
DOI: 10.15252/embr.202256075

Corresponding author(s): Pablo Navarro (pnavarro@pasteur.fr)

Review Timeline:

Transfer from Review Commons:	1st Sep 22
Editorial Decision:	28th Sep 22
Revision Received:	11th Oct 22
Accepted:	14th Oct 22

Editor: Ioannis Papaioannou

Transaction Report: This manuscript was transferred to EMBO Reports following peer review at Review Commons.

Review #1

1. Evidence, reproducibility and clarity:

Evidence, reproducibility and clarity (Required)

Here the authors assess the kinetics of post-mitotic gene reactivation in mouse embryonic stem cells with high temporal resolution. They report a rapid and global reactivation of transcription and suggest that this may differ from other cell types. The fastest reactivating genes are shown to be associated with previously defined CTCF bookmarked sites. However, acute degradation of CTCF did not alter global reactivation kinetics. Nonetheless, the authors identify a small subset of genes which do require CTCF for correct reactivation post-mitosis.

Major comments

1. The authors conclude that their data show that reactivation kinetics in mouse ESCs are distinct from the pattern seen in other cell types, both in the rate of reactivation (faster) and in the order of genes (global vs ubiquitous before cell identity). However, it is not clear to us that mouse ESCs are substantially different in these aspects as the authors emphasise. For example, Kang et al. show that 88% of genes are activated by 60 min, Hsiung et al. indicate that transcriptional re-activation occurs within 40-60 min, whilst Palozola et al. indicate the biggest changes occur between 40-80 min (but do not have further time-points within this window). In the current work, the authors indicate the largest changes occur between 40-60 min which seems comparable to, rather than substantially different from, previous work in other cells. Furthermore, Hsiung et al. indicate that the early transcriptional spike they describe includes both ubiquitous and hematopoietic specific genes; whilst Kang et al. show that bone genes follow the same dynamics as the global pattern of reactivation. These observations would again seem similar to, not substantially different from, observations made in the current work. This either needs to be addressed by modifying the conclusions of the paper, or alternatively the authors should demonstrate and explain more clearly how their data show a distinct pattern from previous work in other cell types. The latter would require re-analysing some of their data in a way which is more directly comparable to the previous work. For example, the authors present their data ranked by the mean of 20-90 min, whereas Hsiung et al. and Palozola et al. order genes and define groups based on when they are first re-activated. Similarly, the groups of genes for GO analysis are defined quite differently. This does not invalidate the current analysis; however, such differences make it very difficult to determine how similar or different the pattern of reactivation is to other cell types.
2. Figures 2A and 2E are described in the text as being ordered by 'increasingly fast reactivation dynamics', which would suggest a ranking based on the time-point at they are reactivated. However, the legend indicates that they are ordered based on the mean of 20-90min, which is related to the amplitude of reactivation, not the time point. This should be revised or clarified in the text. Figure 2E is used to conclude that ESCs are different from other cells where interphasic genes precede cell-type specific genes; however, as indicated above, Figure 2E does not seem to provide any information about the order of activation, only the amplitude. As such, the authors should modify their conclusions or provide

additional evidence to support their statements.

Minor comments

1. In this study, the authors used a double inhibition (CDK1 inhibition followed by nocodazole arrest) as compared to their previous study where they used a nocodazole arrest (Owens et al, Elife, 2019). Inhibition of CDK1 has been shown to change the phosphorylation of many proteins and the chromatin state in ESCs (Michowski et al, Molecular Cell, 2020). Did the authors check whether CDK1 inhibition impacts CTCF binding during mitosis? The authors should also verify CTCF degradation on mitotic chromosomes using these conditions.
2. The authors show that early down-regulated genes in response to CTCF degradation are enriched for previously defined CTCF bookmarked sites and on average show CTCF enrichment at the TSS. However, it is not clear what proportion of these early responders have a promoter associated CTCF peak in mitosis. Is it all of them as suggested in the discussion?
3. The authors show that CTCF bookmarking is only required for reactivation of a small subset of target genes. Could early reactivation of other CTCF bookmarked genes post mitosis be attributed to bookmarking by factors other than CTCF?
4. Could the small number of genes impacted by CTCF degradation in mitosis be a result of cell-to-cell variability in CTCF binding to mitotic chromatin, previously shown by the authors (Owens et al, Elife, 2019)? It would be helpful for the authors to discuss their findings in the context of this previous observation.
5. The final part of the discussion regarding the role of CTCF as a bookmarking factor is difficult to follow and it is not clear that all statements have been directly tested. For example, what is the basis for concluding that bookmarked and non-bookmarked genes reactivate at the same rate? And what is the difference between early responsiveness and rapid reactivation? The authors should attempt to re-word this section to clarify their conclusions.

2. Significance:

Significance (Required)

Compared to previous work, this work provides very high temporal resolution of post-mitotic gene reactivation allowing a detailed description of transcriptional dynamics. This will be of interest to those working in the stem cell and mitotic bookmarking fields. Furthermore, this work tests the role of CTCF in post-mitotic gene reactivation, with the potentially surprising result that most genes are not sensitive to its loss. These findings will be of interest as the field aims to determine the role of mitotic bookmarking factors and define what causes certain genes to be reactivated more rapidly than others.

Keywords for our expertise: chromatin biology, mitotic chromatin, transcriptional regulation

Referees cross-commenting

All of the other reviewer comments seem to be good suggestions and we note there appears to be a good level of agreement and overlap between the reviewer comments.

In particular, we note that reviewer 3 makes a similar point to us about comparisons to previous cell types - their suggestion in major point 2 would be an excellent way to address this. We are also in strong agreement with reviewer 2 that the authors ought to verify CTCF depletion in this context.

We also think that reviewer 2 makes a good point about whether the experiments here really test CTCF bookmarking function. Since CTCF remains depleted, its specific mitotic role cannot be fully distinguished from its early G1 role. However, we also note that depleting and restoring CTCF during mitosis, as suggested by reviewer 2 (if technically possible), could also be difficult to interpret. If restored CTCF is able to rebind to its normal bookmarking sites during the mitotic arrest, what would this mean for the importance of CTCF as a bookmarking factor? It may not be absolutely required throughout mitosis, but it might be important that it is bound before mitotic exit begins (which would still not be distinguished in this new experiment). Although these are very interesting questions to consider, given the likely challenges of these experiments, we would be supportive of the authors addressing this point through re-phrasing/discussing these limitations as reviewer 2 suggests as an alternative to new experiments

3. How much time do you estimate the authors will need to complete the suggested revisions:

Estimated time to Complete Revisions (Required)

(Decision Recommendation)

Between 1 and 3 months

4. Review Commons values the work of reviewers and encourages them to get credit for their work. Select 'Yes' below to register your reviewing activity at Publons; note that the content of your review will not be visible on Publons.

Reviewer Publons

Yes

Review #2

1. Evidence, reproducibility and clarity:

Evidence, reproducibility and clarity (Required)

This interesting manuscript by Chervova and colleagues establish a unique experimental set-up that allows the analysis of gene reactivation in mouse ES cells, upon mitotic exit, with unprecedented

temporal resolution (every 10 min). This analysis provided interesting insights into the complexity of the kinetics of gene re-activation at this stage, with several unique patterns of expression bursts uncovered for different genes.

The authors further show that fast genome activation correlates with CTCF sites, particularly with those bookmarked during mitosis by CTCF. This led to the hypothesis that CTCF could be the major driver for this burst of genome reactivation. Surprisingly, removal of CTCF does not compromise the bulk of genome reactivation although a sub-set of genes (promoter-proximal bookmarked CTCF sites) are sensitive to the absence of CTCF and their re-activation profile changes upon CTCF removal.

The manuscript is technically sound, well executed and of great interest. I am therefore fully supportive of its publication. I would just like to point two major issues that should be addressed.

Major comments:

My major comment relates to fact that despite the technical advances employed (cell sorting & acute depletion strategies), the authors never test directly the "bookmarking" function of CTCF. This is mostly because auxin is kept on the experimental set-up throughout the release phase and hence cells undergo G1 entry without functional CTCF. A bookmarking function would imply that CTCF bound throughout mitosis is required for gene expression upon mitotic exit. To proper test notion, the authors should remove CTCF during mitosis in a reversible manner (in nocodazole arrest), demonstrate that CTCF is transiently removed and efficiently restored (see next point), and only then induce the release with functional CTCF. Only this experimental set-up can test the bonafide CTCF function as a bookmarking TF. Hence, I would suggest that the authors either attempt such experimental approach (that may possibly reveal itself impossible due to the kinetics of CTCF depletion/expression) or alternatively, rephrase the title and manuscript to highlight what is convincingly shown here: that CTCF activity upon mitotic exit controls reactivation of selected genes but not the burst.

Another major comment relates to the fact that the authors never show the efficiency of CTCF depletion in these specific experiments but rather rely on their prior work (and cite the corresponding paper) where they convincingly show the efficacy of the approach. However, relying on historical controls is not a good practice in general and becomes even more important when is to support negative results (e.g. no major changes in G1 burst of transcription upon CTCF depletion). I understand it is virtually impossible to perform a western blot for every single experiment executed, but at least once the authors should demonstrate that the system works as expected. To address this point in a time and cost-effective manner, the authors could perform a paired western-blot and qPCR analysis for selected genes.

Minor comments:

1. Microscopic images in Figure 1B are missing scale bar
2. A small scheme of the experimental layout for the AID experiments would help to visualize how the experiments were performed.

2. Significance:

Significance (Required)

Mitosis imposes major changes in chromatin and a global transcriptional shut-down. After this cell cycle stage, cells need to restore transcription and the mechanisms that control this genome awakening are not fully understood. Gene reactivation upon mitotic exit has been previously investigated in somatic cells in several studies. However, this has not been approached in ES cells at this temporal resolution, and here this analysis is particularly important (and far reaching) for several reasons: first, ES cells have a short G1 so there is an intrinsic demand for fast reactivation at this stage. Secondly, stem cells need to balance their stem cellular stemness with their differentiation capacity, and proper hierarchy of gene re-activation in M-G1 may be directly contribute to this balance.

The major premise for the paper was to test whether CTCF controlled burst of genome activation (much driven by the previously reported mitotic bookmarking activity in these cells), which turned out not to be the case. Yet, I trust the described results, although against this initial premise, are of extreme relevance and will certainly fuel the ongoing controversy on the functional relevance of mitotic bookmarking by transcription factors.

Moreover, the genome-wide description of the dynamics of gene reactivation using their developed system (enabling high temporal resolution) holds the prospect of becoming a reference study for this kinetics analysis at this cell cycle stage. This technical advance is indeed a major asset of the manuscript as analyzing the right cells is key (and perhaps the most challenging) task when aiming to place findings in the context of cell cycle.

Thus, I found the manuscript very significant, timely and of great interest to a broad audience (mitosis, transcription and stem cell fields).

3. How much time do you estimate the authors will need to complete the suggested revisions:

Estimated time to Complete Revisions (Required)

(Decision Recommendation)

Between 1 and 3 months

4. Review Commons values the work of reviewers and encourages them to get credit for their work. Select 'Yes' below to register your reviewing activity at Publons; note that the content of your review will not be visible on Publons.

Reviewer Publons

Yes

Review #3

1. Evidence, reproducibility and clarity:

Evidence, reproducibility and clarity (Required)

Chervova and colleagues have investigated how embryonic stem cells, which undergo very rapid cell cycles, can ensure timely re-activation of (global) gene transcription. By using and RNA-sequencing techniology to specifically detect nascent pre-mRNA molecules, they were able to analyze postmitotic re-activation of gene expression at an unprecedented time resolution of 10 minutes. After establishing very rapid re-activation in ES cells, and identifying sets of genes with specific activation dynamics, they studied the role of CTCF in reactivation of these gene sets. To this end they leveraged their previously published CTCF auxin-induced degradation system. Somewhat surprisingly, CTCF showed only a minor effect on the global transcription dynamics. However some specific genes, notably those which normally bind CTCF proximal to their transcription start site during mitosis, were affected by CTCF depletion.

This manuscript is very well-written and concise. The methodology is robust and appropriate to answer their research questions. Although this study did not reveal any exciting novel insights, the unexpected lack of a major transcription phenotype caused by CTCF depletion is interesting and relevant to the field. Yet I have a couple of suggestions that I feel the authors need to address to strengthen their manuscript further.

Major points:

1. Cell cycle progression in these experiments is poorly characterized. The authors suggest that the quick global reactivation of transcription in ES-cells is required for the rapid start of a new round of DNA replication. As Ctcf bookmarks a subset of genes after mitosis, was there any effect on mitosis exit or S-phase entry when Ctcf is depleted? A time series experiment with EdU labeling in +IAA versus -IAA at various timepoints would help, even if there wouldn't be any difference. If EdU is not feasible, a time series with the CCNA-GFP construct could be an alternative.

1b. In line with this, the authors make a point of histone gene expression as a surrogate for rapid S-phase entry (Figure 1C). Again, I would rather like to see a more direct readout (EdU pulse) to see how long it takes before noticeable S-phase entry.

2. Based on figure 2, the authors conclude that ES-cells display faster, more intense and more widespread compared to other cell types. This is also specifically highlighted in the abstract ("Compared to previous... global reactivation."). However, this claim is difficult to verify for the reader, as the authors do not use more differentiated cell types, and instead cite 3 previously published papers. It would be unreasonable to ask for additional experiments using different cell lines, but I would like to see that the authors re-analyze & plot the data from these three publications as consistently as possible with the current data, and then show these as a Supplementary figure. That may really help the readers to appreciate the relative rapidness and magnitude of this fast burst of transcription in ES cells compared to differentiated cell types.
3. In figure 3, the authors analyze binding of a set of transcription factors to the different groups of genes with various re-activation times. I would like to see a more unsupervised approach to explore which (other) TFs could be enriched in the ultra-rapid reactivating gene group. I understand that these are relevant transcription factors, and that ChIP-sequencing are of mitotic ES cells are unavailable for many transcription factors, but perhaps an in silico prediction approach based on (proximal) TF binding motifs would provide valuable insights.

Minor points:

1. In figure 5A figure legend, please write "yellow shadow area" instead of "shadow area". It wasn't immediately clear to me.
2. In fig 5B the labels LOST and BOOK were non-intuitive to me; consider labeling them with a more informative name, and explaining in a bit more detail I the text why this distinction was made between "bookmarked genes" and "mitotically lost genes".
3. Text describing fig 4A: it should be "shaked-off' instead of "shacked-off".

2. Significance:

Significance (Required)

Although the mitotic bookmarking by CTCF and transcriptional reactivation after G1 is previously published, the authors now describe this process in embryonic stem cells. Since these cells exhibit a very short G1-phase, evaluating the transcriptional reactivation required higher temporal resolution. To tackle this problem, the authors used multiple timepoints short after mitosis and observed very rapid global reactivation of transcription. Although this finding in ES-cells is novel, it mainly builds on similar studies in normal cells and adds little conceptual advances. For example, it is still unclear what mediates the general burst in transcription and what the importance of Ctcf mediated mitotic bookmarking is. Nevertheless, the surprising lack of a major transcription phenotype caused by CTCF depletion is interesting and relevant to the cell cycle field.

Referees cross-commenting

I agree with the other reviewers' feedback.

3. How much time do you estimate the authors will need to complete the suggested revisions:

Estimated time to Complete Revisions (Required)

(Decision Recommendation)

Between 1 and 3 months

4. Review Commons values the work of reviewers and encourages them to get credit for their work. Select 'Yes' below to register your reviewing activity at Publons; note that the content of your review will not be visible on Publons.

Reviewer Publons

Yes

Point by point response to all referees

We thank all three referees for a productive review that has helped us improved our manuscript with several new key experiments and analyses. Below, all comments are reproduced with our responses in blue. All the modifications implemented in the main text are highlighted in yellow.

Reviewer #1 (Evidence, reproducibility and clarity (Required)):

Here the authors assess the kinetics of post-mitotic gene reactivation in mouse embryonic stem cells with high temporal resolution. They report a rapid and global reactivation of transcription and suggest that this may differ from other cell types. The fastest reactivating genes are shown to be associated with previously defined CTCF bookmarked sites. However, acute degradation of CTCF did not alter global reactivation kinetics. Nonetheless, the authors identify a small subset of genes which do require CTCF for correct reactivation post-mitosis.

We thank the referee for an accurate description of our results.

Major comments

1. The authors conclude that their data show that reactivation kinetics in mouse ESCs are distinct from the pattern seen in other cell types, both in the rate of reactivation (faster) and in the order of genes (global vs ubiquitous before cell identity). However, it is not clear to us that mouse ESCs are substantially different in these aspects as the authors emphasise. For example, Kang et al. show that 88% of genes are activated by 60 min, Hsiung et al. indicate that transcriptional re-activation occurs within 40-60 min, whilst Palozola et al. indicate the biggest changes occur between 40-80 min (but do not have further time-points within this window). In the current work, the authors indicate the largest changes occur between 40-60 min which seems comparable to, rather than substantially different from, previous work in other cells. Furthermore, Hsiung et al. indicate that the early transcriptional spike they describe includes both ubiquitous and hematopoietic specific genes; whilst Kang et al. show that bone genes follow the same dynamics as the global pattern of reactivation. These observations would again seem similar to, not substantially different from, observations made in the current work. This either needs to be addressed by modifying the conclusions of the paper, or alternatively the authors should demonstrate and explain more clearly how their data show a distinct pattern from previous work in other cell types. The latter would require re-analysing some of their data in a way which is more directly comparable to the previous work. For example, the authors present their data ranked by the mean of 20-90 min, whereas Hsiung et al. and Palozola et al. order genes and define groups based on when they are first re-activated. Similarly, the groups of genes for GO analysis are defined quite differently. This does not invalidate the current analysis; however, such differences make it very difficult to determine how similar or different the pattern of reactivation is to other cell types.

We agree with the referee that comparisons between studies are difficult to be established, mostly because the metrics used in each study differ substantially. Before describing what we have done to address this point, we would like to clarify the analyses made by each paper; in contrast to the reviewer claim, the other studies do not simply "*order genes and define groups based on when they are first re-activated*":

- in Hsiung et al. they based the analysis on PCA, by ranking genes according to their projection onto the first principal component (PC1), excluding asynchronous cells. They then made three groups according to either the transition from negative to positive values in PC1-projected values (early spike) or arbitrarily upon visual inspection (late plateau and late upregulated). This analysis therefore combines temporal aspects with the shape of the reactivation profile.
- in Palozola et al., they did not rank genes but rather grouped them based on the timepoint at which they increase 1.5-fold above the residual levels measured in mitosis, not considering asynchronous cells. While this is certainly indicative of the first transcriptional events, the behaviour of the genes is largely ignored (early spike, transient activation, progressive increase, etc).
- in Kang et al. they did not rank genes but rather grouped them based on the time point at which their expression exceeds 50% of the average of all time points except asynchronous cells and never went below afterwards. This indicates when genes are consistently activated (ie. transient activations are excluded) but not the reactivation timing per se.

It seems, therefore, that there is no agreement in what to consider early and late reactivated genes among studies, with some focusing more on the shape of the reactivation dynamics (Hsiung et al.) and others either identifying the first transcription events regardless of the behaviour of the genes (Palozola et al.) or considering a gene is reactivated when it reaches a given arbitrary expression level but disregarding strong spike behaviours (Kang et al). We believe all these measurements and criteria are perfectly valid. However, the request of the referee of re-analysing some of our data "in a way which is more directly comparable to the previous work" is not trivial.

We would also like to clarify that we do not base our conclusions on the average of 20-90min post-mitosis (Fig.2A). We apologise if the text was not clear enough regarding this point. In fact, we based our conclusion on the proportion of genes that meet two criteria (Fig.2B): first, the time point at which genes reach or surpass the level measured in asynchronous cells; second, the time point at which genes reach their maximal expression. We believe that these are sensible objective metrics to assess how many genes reactivate rapidly and/or strongly. The text has been, we hope, clarified (page 5: "*To more directly evaluate global reactivation dynamics … substantially higher (Fig. S3B).*").

To address the referee's concern more directly, however, we have now proceeded to plot the data of the other studies with ours, using their own quantifications as provided in the supplementary files of each of these papers but using the two simple metrics we already used in our ms. For the sake of transparency, and as indicated in the acknowledgements of the new version of our ms., we have discussed these analyses with G.Blobel and K.Zaret, senior authors of two of these papers. As shown in Fig.R1, when considering the two metrics used in our original paper (the time at which the maximal transcription is observed and the time at which the levels measured in asynchronous cells are attained or surpassed), it seems clear to us that ES

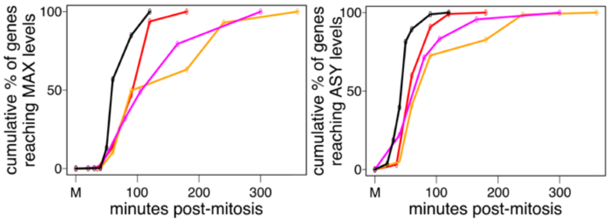

Fig.R1: comparison of our study (black) with published datasets: red, Kang et al.; pink, Palozola et al.; orange, Hsuing et al.

cells' reactivation involves a stronger and faster hypertranscriptional burst for a larger proportion of genes. This data has been added (Fig. S3B) and is briefly commented on page 5: "*direct comparison with previous datasets*[5-7] *indicates than in ES cells it occurs faster and the magnitude and the number of genes engaged appear to be substantially higher (Fig. S3B)*").
Regarding GO associations, previous studies grouped genes according to very different criteria, as indicated above. Despite these differences, both in Palozola et al and in Kang et al they found that genes associated with general functions (cellular organization, cell cycle progression, and RNA/protein metabolism) tend to activate before cell type or developmental genes. In Hsiung et al this was less obvious. In fact, they show the early spike is not very biased to specific processes (see the p-values reported in their Sup.Fig.9), whereas the late upregulating genes were enriched in plasma membrane genes and genes related to erythroid biology (hence displaying some cell type specificity). In our case, we preferred not to group genes for two main reasons because (1) we have a high-resolution temporal analysis and our measurements are very continuous and (2) we observe most genes reactivate very rapidly. Hence, we used a different strategy: first, we used PCA analysis to identify the genes strongly influencing PC1, which captures 80% of the variance of the dataset, and PC2 (12%); then we used these four groups of genes to assess functional categories. As shown in our previous Fig.S2 (now Fig.S4), both top PC1 and bottom PC2 genes, which represent fast reactivated genes (Fig.2D), are enriched in multiple and diverse Terms related to pluripotency, stem cells, the cell cycle and general cellular functions. Our conclusion of a less ordered reactivation in ES cells compared to previous studies is largely based on this analysis; we apologise to the referee if the representation of our previous Fig.2E (now Fig.2F) suggested that we used our ranked heatmap to identify these GO associations. What we did was exclusively for representation purposes: we used all the GO Terms identified through PCA analysis (more than 185 different terms, provided now in a new Supplementary Table S2) to recalculate an FDR for nearly 1000 groups of overlapping genes from top to bottom of the ranked genes heatmap. This gives a more continuous perception of gene reactivation dynamics, showing that all the statistically significant associations derived from the PCA analysis, increasingly characterise genes as they reactivate more rapidly and strongly (see point 2 below). To clarify this, we have now reworded the text on page 6 ("*This suggests that, in contrast to other cell types*[6,7]*… as well as the pluripotency network.*") and added a rearranged version of our previous Fig.S2 (now Fig. S4) as Fig.2E.

2.Figures 2A and 2E are described in the text as being ordered by 'increasingly fast reactivation dynamics', which would suggest a ranking based on the time-point at they are reactivated. However, the legend indicates that they are ordered based on the mean of 20-90min, which is related to the amplitude of reactivation, not the time point. This should be revised or clarified in the text. Figure 2E is used to conclude that ESCs are different from other cells where interphasic genes precede cell-type specific genes; however, as indicated above, Figure 2E does not seem to provide any information about the order of activation, only the amplitude. As such, the authors should modify their conclusions or provide additional evidence to support their statements.

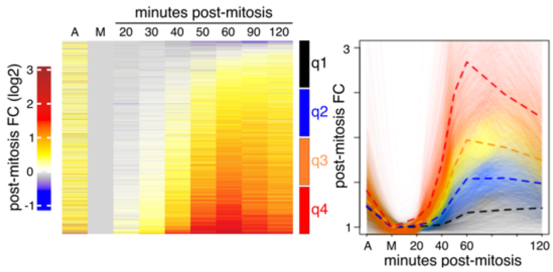

Fig.R2: mean reactivation profile (right) of gene quantiles derived from the heatmap (left) and indicated as q1 (black), q2 (blue), q3 (orange) and q4 (red).

We understand the referee's comment. However, ordering genes by the 20-90min mean does not only

capture the amplitude, but also the timing. To illustrate this, we used the data presented in the heatmap and split the genes in 4 quantiles, from top to bottom, and plotted the mean expression after mitosis (Fig.R2). From q1 to q4, genes reactivate both faster and more strongly. We thought this was already obvious from the heatmap, genes at the top display a level of post-mitotic transcription at 60-120min similar to that observed for genes at the bottom at 30min. To clarify this, we have added the quantile analysis as Fig.S3A and described it on page 5 (“*which orders them according to both the timing and the amplitude of their reactivation (Fig. S3A)”*).

Moreover, to meet the referees concern more directly, we aimed at grouping our genes strictly by reactivation timing, ignoring other parameters and using an approach similar to Palozola et al. based on the time point at which genes reach 1.5-fold levels compared to mitosis. We identified two groups of gees either reactivated within the first 50min after mitosis (early) or afterwards (late). With these two groups of early and late reactivating genes (Fig.R3 top) we interrogated the same GO Terms shown in our previous Fig.S2 related to stem cell biology. We observe that the enrichment is more significant for early genes (Fig.R3 bottom), indicating that regardless of the analysis, ES cells do indeed reactivate a substantial fraction of cell identity genes early on after mitosis. While we have performed these analyses to satisfy the referee, we believe that including them in our manuscript will be a potential source of confusion.

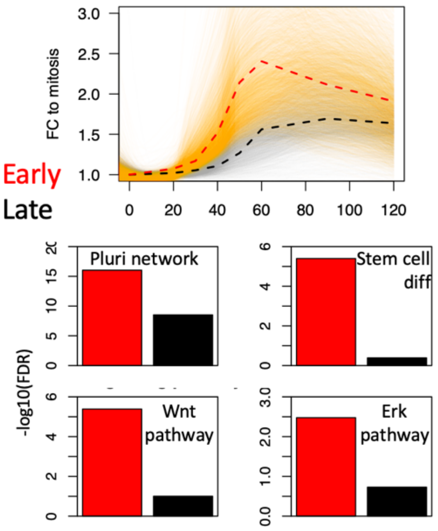

Fig.R3: GO reanalysis after grouping genes as early (red) versus (late) following an approach similar to Palozola et al.

Minor comments

1. In this study, the authors used a double inhibition (CDK1 inhibition followed by nocodazole arrest) as compared to their previous study where they used a nocodazole arrest (Owens et al, Elife, 2019). Inhibition of CDK1 has been shown to change the phosphorylation of many proteins and the chromatin state in ESCs (Michowski et al, Molecular Cell, 2020). Did the authors check whether CDK1 inhibition impacts CTCF binding during mitosis? The authors should also verify CTCF degradation on mitotic chromosomes using these conditions.

We agree that showing that our previously described behaviour of CTCF is reproduced in mitotic cells arrested with a different protocol is important. Therefore, we have performed ChIP-seq of CTCF in asynchronous and in mitotic cells obtained with our double inhibition approach, which confirms mitotic CTCF binding at the regions previously identified in Owens et al (Fig.R4). We have also confirmed by western-blot the effective degradation of CTCF in our double inhibition protocol using CTCF-AID cells (Fig.R4B). This has been added as Fig.S5 and is commented on page 8 (“*After verifying that ES cells synchronised … with or without CTCF (Fig. 4A, Fig. S5C).*”).

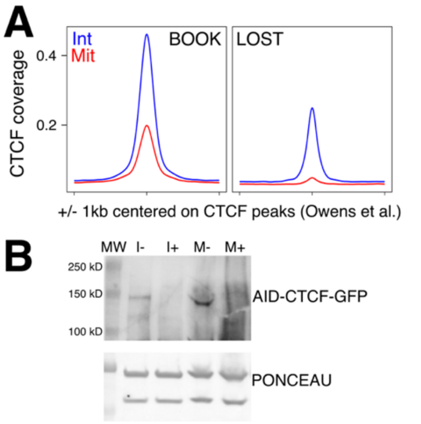

Fig.R4: CTCF controls. (A) ChIP-seq average profile of CTCF at previously identified bookmarked/lost regions, using double inhibition (RO3306 and Nocodazole). (B) Western Blot showing CTCF depletion upon auxin treatment in interphase (I) and mitosis (M)

2. The authors show that early down-regulated genes in response to CTCF degradation are enriched for previously defined CTCF bookmarked sites and on average show CTCF enrichment at the TSS. However, it is not clear what proportion of these early responders have a promoter associated CTCF peak in mitosis. Is it all of them as suggested in the discussion?

We thank the referee for this comment, which points to an important omission in our previous ms. We have now calculated the percentage of CTCF-bound genes, considering a gene as bound when either the promoter (+/-1kb from TSS) or a 25kb-long region centred on the TSS overlaps with a CTCF ChIP-seq peak (Fig.R5). This data confirms the enrichment of bookmarked CTCF peaks at early responders and clarifies that not all but rather 40 to 55% depending on the association criteria are bound by CTCF. These analyses are now included as Fig.S7A and commented on page 9 ("we calculated the percentage of genes… To further expand this analysis, we…").

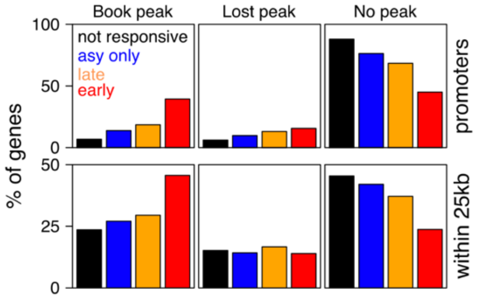

Fig.R5: Percentage of genes displaying CTCF binding in interphase and in mitosis (Book, left panel), in interphase only (Lost, middle panel), or none as a function of its dependency to CTCF: not responsive (black), responsive only in asynchronous cells (blue) or also early (red) or late (orange) after mitosis. Two parameters are shown: binding at the promoter (top) and within a 25kb region spanning the promoter (bottom)

3. The authors show that CTCF bookmarking is only required for reactivation of a small subset of target genes. Could early reactivation of other CTCF bookmarked genes post mitosis be attributed to bookmarking by factors other than CTCF?

We are indeed exploring the possibility that other mitotic bookmarking factors (eg. Esrrb) control gene reactivation after mitosis, both at CTCF bookmarked and non-bookmarked genes. Since we had already mentioned this possibility in the ms ("*Whether other mitotic bookmarking factors display such function and are capable of modulating positively or negatively the post-mitotic burst remains to be directly assessed*"), we believe that no other mention is necessary.

4. Could the small number of genes impacted by CTCF degradation in mitosis be a result of cell-to-cell variability in CTCF binding to mitotic chromatin, previously shown by the authors (Owens et al, Elife, 2019)? It would be helpful for the authors to discuss their findings in the context of this previous observation.

We thank the referee for a careful reading of our previous work. Indeed, we showed that the global retention of CTCF on mitotic chromosomes as visualised by microscopy is quite heterogeneous, particularly in somatic cell lines. Unfortunately, we have not yet investigated the basis of such heterogeneous pattern nor its implications so it is difficult to elaborate on this aspect in the current paper. Moreover, it is also difficult to ascertain whether the heterogenous global retention on mitotic chromosomes reflects heterogeneous mitotic binding at specific sites or not. In this context, we feel that it would be too speculative to link a small transcriptional response upon CTCF depletion to heterogeneous mitotic bookmarking.

5. The final part of the discussion regarding the role of CTCF as a bookmarking factor is difficult to follow and it is not clear that all statements have been directly tested. For example, what is the basis for concluding that bookmarked and non-bookmarked genes reactivate at the

same rate? And what is the difference between early responsiveness and rapid reactivation? The authors should attempt to re-word this section to clarify their conclusions.

The general thinking in the bookmarking field is that bookmarked genes reactivate faster than non-bookmarked genes. However, data supporting this statement is scarce and rather based on correlations. For instance, we provide correlative data in this ms showing that rapidly reactivated genes are in average more frequently associated with mitotic CTCF binding. However, the opposite is not necessarily true: if we compare the reactivation dynamics of genes bookmarked by CTCF to genes where CTCF binds exclusively in interphase, we observe similar reactivation profiles (Fig.R6). This comparison, which we believe is more informative – albeit also correlative –, does not support the notion of CTCF bookmarking as an accelerator of post-mitotic gene reactivation. We have therefore added this data as Fig.S7B, in support of our statement on page 12, which has been further clarified ("*Indeed, while rapidly reactivated… similar reactivation profiles (Fig. S7B).*").

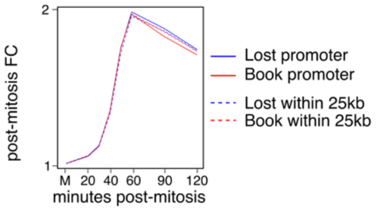

Fig.R6: average post-mitotic dynamics of genes bound by CTCF either in interphase and in mitosis (Book, red) or only in interphase (Lost, blue). Two parameters are shown: binding at the promoter (solid lines) and within a 25kb region spanning the promoter (dashed lines)

Does this mean mitotic bookmarking by CTCF makes no difference in terms of time of reactivation? To answer this question, we wanted to introduce a distinction between rapid reactivation per se and early responsiveness: the former implies that CTCF bookmarked genes reactivate faster than genes bound by CTCF exclusively in interphase; the latter that CTCF bookmarking confers early dependency (responsiveness) to CTCF. Indeed, we showed already in the original manuscript that among CTCF-responsive genes, those that depend on CTCF immediately after mitosis are more frequently bookmarked by CTCF than those that are bound by CTCF only in interphase. We hope that the modifications implemented above will help clarify this issue.

Reviewer #1 (Significance (Required)):

Compared to previous work, this work provides very high temporal resolution of post-mitotic gene reactivation allowing a detailed description of transcriptional dynamics. This will be of interest to those working in the stem cell and mitotic bookmarking fields. Furthermore, this work tests the role of CTCF in post-mitotic gene reactivation, with the potentially surprising result that most genes are not sensitive to its loss. These findings will be of interest as the field aims to determine the role of mitotic bookmarking factors and define what causes certain genes to be reactivated more rapidly than others.

Keywords for our expertise: chromatin biology, mitotic chromatin, transcriptional regulation

We thank the referee for this comment.

Referees cross-commenting

All of the other reviewer comments seem to be good suggestions and we note there appears to be a good level of agreement and overlap between the reviewer comments.

In particular, we note that reviewer 3 makes a similar point to us about comparisons to previous cell types - their suggestion in major point 2 would be an excellent way to address this. We are also in strong agreement with reviewer 2 that the authors ought to verify CTCF depletion in this context.

We agree and have therefore used the proposed analysis made by Reviewer 3 to address point 1 of Reviewer 1. CTCF depletion has now been confirmed.

We also think that reviewer 2 makes a good point about whether the experiments here really test CTCF bookmarking function. Since CTCF remains depleted, its specific mitotic role cannot be fully distinguished from its early G1 role. However, we also note that depleting and restoring CTCF during mitosis, as suggested by reviewer 2 (if technically possible), could also be difficult to interpret. If restored CTCF is able to rebind to its normal bookmarking sites during the mitotic arrest, what would this mean for the importance of CTCF as a bookmarking factor? It may not be absolutely required throughout mitosis, but it might be important that it is bound before mitotic exit begins (which would still not be distinguished in this new experiment). Although these are very interesting questions to consider, given the likely challenges of these experiments, we would be supportive of the authors addressing this point through re-phrasing/discussing these limitations as reviewer 2 suggests as an alternative to new experiments

We agree with Reviewer 1 and thank him/her to further elaborate on the problems posed by the experiment suggested by Reviewer 2.

Reviewer #2 (Evidence, reproducibility and clarity (Required)):

This interesting manuscript by Chervova and colleagues establish a unique experimental set-up that allows the analysis of gene reactivation in mouse ES cells, upon mitotic exit, with unprecedented temporal resolution (every 10 min). This analysis provided interesting insights into the complexity of the kinetics of gene re-activation at this stage, with several unique patterns of expression bursts uncovered for different genes.
The authors further show that fast genome activation correlates with CTCF sites, particularly with those bookmarked during mitosis by CTCF. This led to the hypothesis that CTCF could be the major driver for this burst of genome reactivation. Surprisingly, removal of CTCF does not compromise the bulk of genome reactivation although a sub-set of genes (promoter-proximal bookmarked CTCF sites) are sensitive to the absence of CTCF and their re-activation profile changes upon CTCF removal.

The manuscript is technically sound, well executed and of great interest. I am therefore fully supportive of its publication. I would just like to point two major issues that should be addressed.

We thank the referee for such a positive evaluation of our work and acknowledging its importance.

Major comments:
My major comment relates to fact that despite the technical advances employed (cell sorting & acute depletion strategies), the authors never test directly the "bookmarking" function of CTCF. This is mostly because auxin is kept on the experimental set-up throughout the release phase and hence cells undergo G1 entry without functional CTCF. A bookmarking function would imply that CTCF bound throughout mitosis is required for gene expression upon mitotic exit. To proper test notion, the authors should remove CTCF during mitosis in a reversible manner (in nocodazole arrest), demonstrate that CTCF is transiently removed and efficiently restored (see next point), and only then induce the release with functional CTCF. Only this experimental set-up can test the bonafide CTCF function as a bookmarking TF. Hence, I would suggest that the authors either attempt such experimental approach (that may possibly reveal itself impossible due to the kinetics of CTCF depletion/expression) or alternatively, rephrase the title and manuscript to highlight what is convincingly shown here: that CTCF activity upon mitotic exit controls reactivation of selected genes but not the burst.

We agree with this referee and also with Reviewer 1 comment on this point. The proposed experiment is hardly doable and the interpretations would also be complicated without a close examination of the timing of rebinding following re-entry into interphase. This being said, we have to acknowledge that should the impact of CTCF had been more penetrant, with a strong and large effect across many genes, then we would certainly have attempted executing this key experiment. Given the mild impact of CTCF we feel the effort is not justified and have therefore preferred to: 1/ rephrase the title as suggested by the reviewer: "*CTCF controls selected genes subject to mitotic bookmarking during the fast post-mitotic genome reactivation of ES cells*" and 2/ tone down the conclusions (e.g. On page 3: "*providing a direct experimental evidence of the* ***function of CTCF at the mitosis to G1 transition***").

Another major comment relates to the fact that the authors never show the efficiency of CTCF depletion in these specific experiments but rather rely on their prior work (and cite the corresponding paper) where they convincingly show the efficacy of the approach. However, relying on historical controls is not a good practice in general and becomes even more important when is to support negative results (e.g. no major changes in G1 burst of transcription upon CTCF depletion). I understand it is virtually impossible to perform a western blot for every single experiment executed, but at least once the authors should demonstrate that the system works as expected. To address this point in a time and cost-effective manner, the authors could perform a paired western-blot and qPCR analysis for selected genes.

For one of the three kinetics presented in the original manuscript we had prepared samples for western-blot. Results are now provided (Fig.R4B) in our responses to Reviewer 1.

Minor comments:
1. Microscopic images in Figure 1B are missing scale bar

This has been implemented.

2. A small scheme of the experimental layout for the AID experiments would help to visualize how the experiments were performed.

We thank the referee for this suggestion, a scheme has been added to Fig.4.

Reviewer #2 (Significance (Required)):

Mitosis imposes major changes in chromatin and a global transcriptional shut-down. After this cell cycle stage, cells need to restore transcription and the mechanisms that control this genome awakening are not fully understood. Gene reactivation upon mitotic exit has been previously investigated in somatic cells in several studies. However, this has not been approached in ES cells at this temporal resolution, and here this analysis is particularly important (and far reaching) for several reasons: first, ES cells have a short G1 so there is an intrinsic demand for fast reactivation at this stage. Secondly, stem cells need to balance their stem cellular stemness with their differentiation capacity, and proper hierarchy of gene re-activation in M-G1 may be directly contribute to this balance.
The major premise for the paper was to test whether CTCF controlled burst of genome activation (much driven by the previously reported mitotic bookmarking activity in these cells), which turned out not to be the case. Yet, I trust the described results, although against this initial premise, are of extreme relevance and will certainly fuel the ongoing controversy on the functional relevance of mitotic bookmarking by transcription factors.
Moreover, the genome-wide description of the dynamics of gene reactivation using their developed system (enabling high temporal resolution) holds the prospect of becoming a reference study for this kinetics analysis at this cell cycle stage. This technical advance is indeed a major asset of the manuscript as analyzing the right cells is key (and perhaps the most challenging) task when aiming to place findings in the context of cell cycle.
Thus, I found the manuscript very significant, timely and of great interest to a broad audience (mitosis, transcription and stem cell fields).

We thank the referee for an extremely positive evaluation of our work.

Reviewer #3 (Evidence, reproducibility and clarity (Required)):

Chervova and colleagues have investigated how embryonic stem cells, which undergo very rapid cell cycles, can ensure timely re-activation of (global) gene transcription. By using and RNA-sequencing techniology to specifically detect nascent pre-mRNA molecules, they were able to analyze postmitotic re-activation of gene expression at an unprecedented time resolution of 10 minutes. After establishing very rapid re-activation in ES cells, and identifying sets of genes with specific activation dynamics, they studied the role of CTCF in reactivation of these gene sets. To this end they leveraged their previously published CTCF auxin-induced degradation system. Somewhat surprisingly, CTCF showed only a minor effect on the global transcription dynamics. However, some specific genes, notably those which normally bind CTCF proximal to their transcription start site during mitosis, were affected by CTCF depletion. This manuscript is very well-written and concise. The methodology is robust and appropriate to answer their research questions. Although this study did not reveal any exciting novel insights, the unexpected lack of a major transcription phenotype caused by CTCF depletion is interesting and relevant to the field. Yet I have a couple of suggestions that I feel the authors need to address to strengthen their manuscript further.

We acknowledge the referee for a positive evaluation of our work and manuscript.

Major points:

1. Cell cycle progression in these experiments is poorly characterized. The authors suggest that the quick global reactivation of transcription in ES-cells is required for the rapid start of a new round of DNA replication. As Ctcf bookmarks a subset of genes after mitosis, was there any effect on mitosis exit or S-phase entry when Ctcf is depleted? A time series experiment with EdU labeling in +IAA versus -IAA at various timepoints would help, even if there wouldn't be any difference. If EdU is not feasible, a time series with the CCNA-GFP construct could be an alternative.

We thank the referee for this comment, which suggests that some of our text can be misleading. Indeed, we did not aim at suggesting that "the quick global reactivation of transcription in ES-cells is required for the rapid start of a new round of DNA replication". Rather, the only idea we wanted to convey is perfectly summarised by Reviewer 2 in the summary made of our work ("ES cells have a short G1 so there is an intrinsic demand for fast reactivation at this stage"). We have therefore used Reviewer 2 wording to describe this aspect and avoid this confusion by any reader (page 2).

Building on this point, the referee asks whether CTCF may not be required to elicit fast replication. While this question is of interest, we believe there is already strong evidence in the literature showing this is not the case. Indeed, previous papers have shown a lack of cell cycle issues in the same mutant cells after even several full days of CTCF depletion (eg. Nora et al. 2017; PMID: 28525758). This strongly indicates that the impact on mitotic exit or S-phase entry in our experimental setup would be, if any, extremely subtle. In agreement, analysis of several markers indicates cell cycle genes are similarly expressed in the presence and absence of CTCF (FigR.7).

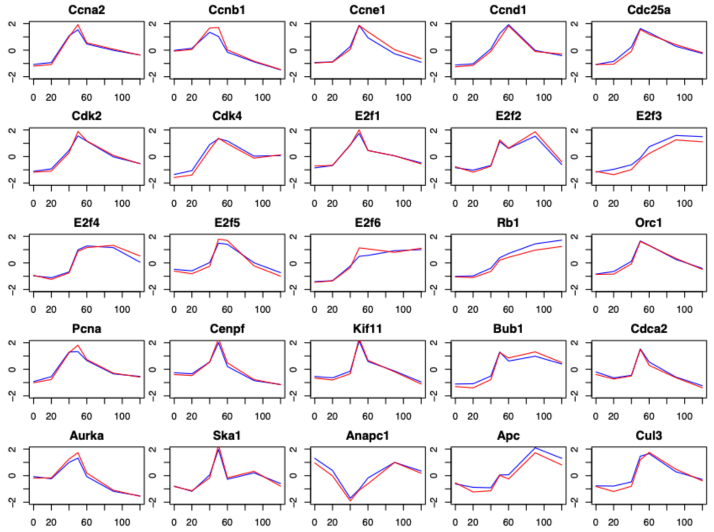

Fig.R7: post-mitotic transcription dynamics of cell cycle regulators in the presence (blue) and absence (red) of CTCF

1b. In line with this, the authors make a point of histone gene expression as a surrogate for rapid S-phase entry (Figure 1C). Again, I would rather like to see a more direct readout (EdU pulse) to see how long it takes before noticeable S-phase entry.

We agree with the referee that histone gene expression is too indirect. We have now performed an EdU pulse experiment (Fig.R8) to visualise by microscopy that the first replication events after release from a mitotic

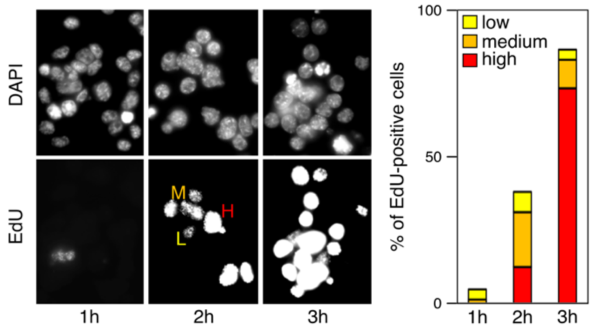

Fig.R8: results of EdU labelling quantifying replicating cells 1, 2 or 3 hours after mitosis.

blockade start between 60 and 120min, with around 80% of cells displaying robust replication activity by 180min. Moreover, compellingly illustrating the intrinsic need for fast reactivation in ES cells, despite experimental manipulations, replication can be observed starting as early as 1h after release from metaphase block. This data has been added to the new version of the ms. as Fig.S2, referenced page 5 ("*Nevertheless, all histone genes showed … matching a remarkably fast entry into S phase, which could be observed as early as 1h after division by EdU incorporation (Fig. S2).*").

2. Based on figure 2, the authors conclude that ES-cells display faster, more intense and more widespread compared to other cell types. This is also specifically highlighted in the abstract ("Compared to previous... global reactivation."). However, this claim is difficult to verify for the reader, as the authors do not use more differentiated cell types, and instead cite 3 previously published papers. It would be unreasonable to ask for additional experiments using different cell lines, but I would like to see that the authors re-analyze & plot the data from these three publications as consistently as possible with the current data, and then show these as a Supplementary figure. That may really help the readers to appreciate the relative rapidness and magnitude of this fast burst of transcription in ES cells compared to differentiated cell types.

We thank this referee for this comment, which we believe is now fully addressed in our response to the first point of Reviewer 1 (Fig.R1): we have plotted the data of the cited papers in parallel to ours to show that, indeed, in ES cells the proportion of genes reaching their maximal transcription or reaching/surpassing the levels measured in asynchronous cells increases faster and involves more genes than in other cell types.

3. In figure 3, the authors analyze binding of a set of transcription factors to the different groups of genes with various re-activation times. I would like to see a more unsupervised approach to explore which (other) TFs could be enriched in the ultra-rapid reactivating gene group. I understand that these are relevant transcription factors, and that ChIP-sequencing are of mitotic ES cells are unavailable for many transcription factors, but perhaps an in silico prediction approach based on (proximal) TF binding motifs would provide valuable insights.

We fully agree with the reviewer; we are currently working along these lines. One of our best candidates is c-Myc, which we already mentioned in the discussion of our previous version of the ms. While it would not be reasonable for us to fully reveal our unpublished results in this area, we are now showing (Fig.R9) that the cMyc motif is prevalent at the promoter of fast/strong reactivating genes and MYC ChIP-seq peaks found enriched based on available publications (Fig.R9), supporting the suggestion we already made in the discussion. This data has been introduced as Fig.S8, referenced on page 10.

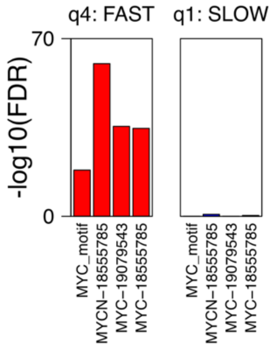

Fig.R9: statistical enrichments of two groups of post-mitotic reactivation genes (fast and slow, corresponding to q4 and q1 in figR2). The analysed parameters were the presence of the MYC motif at the TSS, or the presence of MYC ChIP-seq peaks in PMID18555785, PMID19079543, PMID18555785.

Minor points:

1. In figure 5A figure legend, please write "yellow shadow area" instead of "shadow area". It wasn't immediately clear to me.
This has been implemented.

2. In fig 5B the labels LOST and BOOK were non-intuitive to me; consider labeling them with a more informative name, and explaining in a bit more detail I the text why this distinction was made between "bookmarked genes" and "mitotically lost genes".
The referee may have missed that this nomenclature was defined at an earlier stage of the manuscript: *"Since CTCF has been shown to display robust mitotic binding at a large subset of its interphase targets in ES cells11, we further distinguished between genes associated with CTCF during mitosis or only during interphase (bookmarked and lost, respectively, in Fig. 3D)."*.

3. Text describing fig 4A: it should be "shaked-off' instead of "shacked-off".
This has been implemented.

Reviewer #3 (Significance (Required)):

Although the mitotic bookmarking by CTCF and transcriptional reactivation after G1 is previously published, the authors now describe this process in embryonic stem cells. Since these cells exhibit a very short G1-phase, evaluating the transcriptional reactivation required higher temporal resolution. To tackle this problem, the authors used multiple timepoints short after mitosis and observed very rapid global reactivation of transcription. Although this finding in ES-cells is novel, it mainly builds on similar studies in normal cells and adds little conceptual advances. For example, it is still unclear what mediates the general burst in transcription and what the importance of Ctcf mediated mitotic bookmarking is. Nevertheless, the surprising lack of a major transcription phenotype caused by CTCF depletion is interesting and relevant to the cell cycle field.
We hope that, after having more directly supported the observation that ES cells reactivate their genome with unprecedented efficiency compared to somatic cells, this referee will find the paper adds increased conceptual advance beyond the lack of effects of CTCF depletion. We believe, indeed, that the fast reactivation dynamics of ES cells is also important for the fields of stem cell and developmental biology.

Referees cross-commenting
I agree with the other reviewers' feedback.

1st Editorial Decision **28th Sep 2022**

Dear Dr. Navarro,

Thank you for the submission of your revised manuscript to EMBO reports through Review Commons. We have now received the full set of reports from the three referees that were asked to re-evaluate your study. Please find their reports below.

As you will see, the referees find that the study was significantly improved during revision and recommend publication. They request only few minor changes in the text, which I need you to address before I can accept the manuscript.

From the editorial side, there are also a few things that we need before we can proceed with the official acceptance of your manuscript:

- Please include in your submission a .docx formatted version of your revised manuscript (including legends for main figures, EV figures and tables). Please make sure that the changes are highlighted (or use the "track changes" tool) to be clearly visible.

- You can either publish the study as a short report or as a full article. For short reports, the revised manuscript should not exceed 27,000 characters (including spaces but excluding materials & methods and references) and 5 main plus 5 expanded view figures. The results and discussion sections must further be combined, which will help to shorten the manuscript text by eliminating some redundancy that is inevitable when discussing the same experiments twice. For a normal article there are no length limitations, but it should have more than 5 main figures and the results and discussion sections must be separate. In both cases, the entire materials and methods must be included in the main manuscript file.

- Please provide up to 5 keywords in your revised manuscript.

- The title should be short (up to 100 characters including spaces) and informative, and it should not contain any abbreviations. The abstract should be a single paragraph describing all key novel findings of the study, written in present tense, and it should not exceed 175 words. Please revise both accordingly.

- Your Figure legends have been inspected by our data editors for completeness and accuracy. Please see the required changes in the attached Word file.

- The RNA sequencing dataset produced in this study should be listed in a formal "Data Availability " section (placed after Materials & Methods) that follows the model below (see also).

Data availability

The dataset produced in this study is available in the following database:

- RNA-Seq data: Gene Expression Omnibus GSE46843 (https://www.ncbi.nlm.nih.gov/geo/query/acc.cgi?acc=GSE46843)

- Please update the 'Conflict of interest' paragraph to our new 'Disclosure and competing interests statement'. For more information see
https://www.embopress.org/page/journal/14693178/authorguide#conflictsofinterest

- We now use CRediT to specify the contributions of each author in the journal submission system. CRediT replaces the author contribution section, which should be removed from the manuscript. Please use the free text box to provide more detailed descriptions. See also guide to authors:
.

- Please note our reference format and update the list of references accordingly:
.

- We also need a completed author checklist, which you can download from our author guidelines (). Please insert information in the checklist that is also reflected in the manuscript. The completed author checklist will be part of the Review Process File (RPF); please see below for more information.

- Figure callouts for Fig S2A&B and S6A&B are missing, please make sure that all panels are called out in your revised manuscript.

- Tables S1-S3 should be named Dataset EV1-EV3, and the legends should be included in the corresponding files in a separate sheet. See detailed instructions regarding expanded view here:

- We have replaced Supplementary Information with Expanded View (EV) Figures and Tables that are collapsible/expandable online. A maximum of 5 EV Figures can be typeset. EV Figures should be cited as 'Figure EV1, Figure EV2" etc... in the text and their respective legends should be included in the main text after the legends of regular figures. For the figures that you do NOT wish to display as Expanded View figures, they should be bundled together with their legends in a single PDF file called *Appendix*, which should start with a short Table of Content (TOC). Appendix figures should be referred to in the main text as: "Appendix Figure S1, Appendix Figure S2" etc. The names in the TOC also need to be "Appendix Figure S1", "Appendix Figure S1" etc.
Please remove table legends from the Appendix file.

If you wish to promote up to 5 supplementary figures to the Expanded View format you can do so, but it is also possible to keep all supplementary figures in the new Appendix file.

- "Methods" needs correcting to "Materials and Methods".

- Figure legends should be placed after the Reference list.

- Please note that EMBO press papers are accompanied online by
A) a short (1-2 sentences) summary of the findings and their significance,
B) 2-3 bullet points highlighting key results, and
C) a synopsis image that is exactly 550 pixels wide and 200-600 pixels high (the height is variable). You can either show a model or key data in the synopsis image. Please note that text needs to be readable at the final size.
Please send us this information along with your revised manuscript.

- As part of the EMBO publications' Transparent Editorial Process, EMBO reports publishes online a Review Process File to accompany accepted manuscripts. This File will be published in conjunction with your paper and will include the referee reports, your point-by-point response and all pertinent correspondence relating to the manuscript.

You can opt out of this by letting the editorial office know (emboreports@embo.org). If you do opt out, the Review Process File link will point to the following statement: "No Review Process File is available with this article, as the authors have chosen not to make the review process public in this case."

We would also welcome the submission of cover suggestions, or motifs to be used by our Graphics Illustrator in designing a cover.

We look forward to seeing a final version of your manuscript as soon as possible.

Yours sincerely,

Ioannis Papaioannou, PhD
Editor
EMBO reports

Referee #1:

I have carefully read the revised manuscript and the point-by-point answer to our previous concerns. The authors have appropriately addressed all points and strengthened the manuscript substantially. I particularly appreciate their effort to re-analyze the previously published datasets (Figure S3B).

I only spotted one typo, at the end of page 5 "...indicates than in ES cells...". "than" should be "that".

Referee #2:

In this revised version of the manuscript, the authors have satisfactorily addressed most of my comments on the manuscript. I also trust that the current version addresses the major points raised by the other referees. I can therefore support its publication in EMBO Reports.

As a side note to my first major comment, I agree with the authors' assessment (reiterated by reviewer #1 in the cross-commenting section) that given the limited effect of CTCF and the complicated nature of the required experiments, the attempt to test the true "bookmarking function" using fast restoration experiments may not be worth the effort. The alternative path chosen by the authors, which concentrated on toning down the bookmarking effect, does appropriately address my concern and the manuscript now better describes the stronger points made here.

Referee #3:

The authors have worked hard to carefully address our comments and we appreciate the effort made to respond to our suggestions, and to clearly highlight the changes made in the manuscript. We agree that the manuscript has been improved, and in particular the analysis added as FigS3B strengthens one of their key conclusions about rapid reactivation in comparison to other cell types. This work is of high quality and will be of interest to the stem cell and mitotic bookmarking fields and we would be strongly supportive of publication of the manuscript in its current form, with two minor comments:

1. We suggest being more cautious in the results section when drawing conclusions about differences between ESCs and other cell types for GO term enrichment. As the authors acknowledge in their response, these analyses were performed quite differently and a direct comparison has not been made. The data strongly support their conclusion that ESCs rapidly reactivate both cell type-specific and general genes; however, comparisons with other cell types might be better placed in the discussion, or at least tempered/caveated slightly.
2. Ensure methods are updated to include details of new experiments/analysis performed during revision.

Point by point response to all referees

We thank the referees for their work and productive comments, which have helped us improve our paper.

Referee #1:

I have carefully read the revised manuscript and the point-by-point answer to our previous concerns. The authors have appropriately addressed all points and strengthened the manuscript substantially. I particularly appreciate their effort to re-analyze the previously published datasets (Figure S3B).

I only spotted one typo, at the end of page 5 "...indicates than in ES cells...". "than" should be "that".

We agree that the direct comparison with previous datasets is important, beyond its limitations. The sentence with the typo spotted by the referee has been changed to meet a comment made by Referee 3.

Referee #2:

In this revised version of the manuscript, the authors have satisfactorily addressed most of my comments on the manuscript. I also trust that the current version addresses the major points raised by the other referees. I can therefore support its publication in EMBO Reports.

As a side note to my first major comment, I agree with the authors' assessment (reiterated by reviewer #1 in the cross-commenting section) that given the limited effect of CTCF and the complicated nature of the required experiments, the attempt to test the true "bookmarking function" using fast restoration experiments may not be worth the effort. The alternative path chosen by the authors, which concentrated on toning down the bookmarking effect, does appropriately address my concern and the manuscript now better describes the stronger points made here.

We agree the paper now gives a more balanced view on mitotic bookmarking.

Referee #3:

The authors have worked hard to carefully address our comments and we appreciate the effort made to respond to our suggestions, and to clearly highlight the changes made in the manuscript. We agree that the manuscript has been improved, and in particular the analysis added as FigS3B strengthens one of their key conclusions about rapid reactivation in comparison to other cell types. This work is of high quality and will be of interest to the stem cell and mitotic bookmarking fields and we would be strongly supportive of publication of the manuscript in its current form, with two minor comments:

1. We suggest being more cautious in the results section when drawing conclusions about differences between ESCs and other cell types for GO term enrichment. As the authors acknowledge in their response, these analyses were performed quite differently and a direct

comparison has not been made. The data strongly support their conclusion that ESCs rapidly reactivate both cell type-specific and general genes; however, comparisons with other cell types might be better placed in the discussion, or at least tempered/caveated slightly.
2. Ensure methods are updated to include details of new experiments/analysis performed during revision.

We have now slightly modified the results section to explicitly alert the reader that the direct comparison between datasets needs to be taken cautiously ("*Since this intense burst of transcriptional activity has been previously observed (Hsiung et al, 2016; Palozola et al, 2017; Kang et al, 2020),* ***we aimed at directly comparing these results with previous datasets, even though intrinsic experimental differences may not allow to reach definitive conclusions.*** *Our comparisons indicate that …*").

We have included the parts that were missing in the methods section (such as EdU labelling and CTCF ChIP-seq).

Pablo Navarro
Pasteur Institute
Developmental and Stem Cell Biology
25-28 Rue de docteur Roux
Paris, none 75015
France

Dear Dr. Navarro,

I am very pleased to accept your manuscript for publication in the next available issue of EMBO reports. Thank you for your contribution to our journal.

At the end of this email I include important information about how to proceed. Please ensure that you take the time to read the information and complete and return the necessary forms to allow us to publish your manuscript as quickly as possible.

As part of the EMBO publication's Transparent Editorial Process, EMBO reports publishes online a Review Process File to accompany accepted manuscripts. As you are aware, this File will be published in conjunction with your paper and will include the referee reports, your point-by-point response and all pertinent correspondence relating to the manuscript.

If you do NOT want this File to be published, please inform the editorial office within 2 days, if you have not done so already, otherwise the File will be published by default [contact: emboreports@embo.org]. If you do opt out, the Review Process File link will point to the following statement: "No Review Process File is available with this article, as the authors have chosen not to make the review process public in this case."

Should you be planning a Press Release on your article, please get in contact with emboreports@wiley.com as early as possible, in order to coordinate publication and release dates.

Thank you again for your contribution to EMBO reports and congratulations on a successful publication. Please consider us again in the future for your most exciting work.

Yours sincerely,

Ioannis Papaioannou, PhD
Editor
EMBO reports

****

THINGS TO DO NOW:

You will receive proofs by e-mail approximately 2-3 weeks after all relevant files have been sent to our Production Office; you should return your corrections within 2 days of receiving the proofs.

EMBO Press participates in many Publish and Read agreements that allow authors to publish Open Access with reduced/no publication charges. Check your eligibility: https://authorservices.wiley.com/author-resources/Journal-Authors/open-access/affiliation-policies-payments/index.html

Please inform us if there is likely to be any difficulty in reaching you at the above address at that time. Failure to meet our deadlines may result in a delay of publication, or publication without your corrections.

All further communications concerning your paper should quote reference number EMBOR-2022-56075V2 and be addressed to emboreports@wiley.com.

Should you be planning a Press Release on your article, please get in contact with emboreports@wiley.com as early as possible, in order to coordinate publication and release dates.

EMBO Press Author Checklist

Corresponding Author Name: Navarro	
Journal Submitted to: EMBO Reports	
Manuscript Number: EMBOR-2022-56075V1	

USEFUL LINKS FOR COMPLETING THIS FORM
The EMBO Journal - Author Guidelines
EMBO Reports - Author Guidelines
Molecular Systems Biology - Author Guidelines
EMBO Molecular Medicine - Author Guidelines

Reporting Checklist for Life Science Articles (updated January 2022)
This checklist is adapted from Materials Design Analysis Reporting (MDAR) Checklist for Authors. MDAR establishes a minimum set of requirements in transparent reporting in the life sciences (see Statement of Task: 10.31222/osf.io/9sm4x). Please follow the journal's guidelines in preparing your manuscript.
Please note that a copy of this checklist will be published alongside your article.

Abridged guidelines for figures
1. Data
The data shown in figures should satisfy the following conditions:
- the data were obtained and processed according to the field's best practice and are presented to reflect the results of the experiments in an accurate and unbiased manner.
- ideally, figure panels should include only measurements that are directly comparable to each other and obtained with the same assay.
- plots include clearly labeled error bars for independent experiments and sample sizes. Unless justified, error bars should not be shown for technical replicates.
- if n<5, the individual data points from each experiment should be plotted. Any statistical test employed should be justified.
- Source Data should be included to report the data underlying figures according to the guidelines set out in the authorship guidelines on Data Presentation.

2. Captions
Each figure caption should contain the following information, for each panel where they are relevant:
- a specification of the experimental system investigated (eg cell line, species name).
- the assay(s) and method(s) used to carry out the reported observations and measurements.
- an explicit mention of the biological and chemical entity(ies) that are being measured.
- an explicit mention of the biological and chemical entity(ies) that are altered/varied/perturbed in a controlled manner.
- the exact sample size (n) for each experimental group/condition, given as a number, not a range;
- a description of the sample collection allowing the reader to understand whether the samples represent technical or biological replicates (including how many animals, litters, cultures, etc.).
- a statement of how many times the experiment shown was independently replicated in the laboratory.
- definitions of statistical methods and measures:
  - common tests, such as t-test (please specify whether paired vs. unpaired), simple χ2 tests, Wilcoxon and Mann-Whitney tests, can be unambiguously identified by name only, but more complex techniques should be described in the methods section;
  - are tests one-sided or two-sided?
  - are there adjustments for multiple comparisons?
  - exact statistical test results, e.g., P values = x but not P values < x;
  - definition of 'center values' as median or average;
  - definition of error bars as s.d. or s.e.m.

Please complete ALL of the questions below.
Select "Not Applicable" only when the requested information is not relevant for your study.

Materials

Newly Created Materials	Information included in the manuscript?	In which section is the information available? (Reagents and Tools Table, Materials and Methods, Figures, Data Availability Section)
New materials and reagents need to be available; do any restrictions apply?	Not Applicable	

Antibodies	Information included in the manuscript?	In which section is the information available? (Reagents and Tools Table, Materials and Methods, Figures, Data Availability Section)
For **antibodies** provide the following information:<br>- Commercial antibodies: RRID (if possible) or supplier name, catalogue number and or/clone number<br>- Non-commercial: RRID or citation	Yes	Material and Methods

DNA and RNA sequences	Information included in the manuscript?	In which section is the information available? (Reagents and Tools Table, Materials and Methods, Figures, Data Availability Section)
**Short novel DNA or RNA including primers, probes**: provide the sequences.	Not Applicable	

Cell materials	Information included in the manuscript?	In which section is the information available? (Reagents and Tools Table, Materials and Methods, Figures, Data Availability Section)
**Cell lines:** Provide species information, strain. Provide accession number in repository **OR** supplier name, catalog number, clone number, and/**OR** RRID.	Yes	Material and Methods
**Primary cultures:** Provide species, strain, sex of origin, genetic modification status.	Not Applicable	
Report if the cell lines were recently **authenticated** (e.g., by STR profiling) and tested for mycoplasma contamination.	Yes	Material and Methods

Experimental animals	Information included in the manuscript?	In which section is the information available? (Reagents and Tools Table, Materials and Methods, Figures, Data Availability Section)
**Laboratory animals or Model organisms:** Provide species, strain, sex, age, genetic modification status. Provide accession number in repository **OR** supplier name, catalog number, clone number, **OR** RRID.	Not Applicable	
**Animal observed in or captured from the field:** Provide species, sex, and age where possible.	Not Applicable	
Please detail **housing and husbandry conditions**.	Not Applicable	

Plants and microbes	Information included in the manuscript?	In which section is the information available? (Reagents and Tools Table, Materials and Methods, Figures, Data Availability Section)
**Plants:** provide species and strain, ecotype and cultivar where relevant, unique accession number if available, and source (including location for collected wild specimens).	Not Applicable	
**Microbes:** provide species and strain, unique accession number if available, and source.	Not Applicable	

Human research participants	Information included in the manuscript?	In which section is the information available? (Reagents and Tools Table, Materials and Methods, Figures, Data Availability Section)
If collected and within the bounds of privacy constraints report on age, sex and gender or ethnicity for all study participants.	Not Applicable	

Core facilities	Information included in the manuscript?	In which section is the information available? (Reagents and Tools Table, Materials and Methods, Figures, Data Availability Section)
If your work benefited from core facilities, was their service mentioned in the acknowledgments section?	Yes	Acknowledgments

Design

Study protocol	Information included in the manuscript?	In which section is the information available? (Reagents and Tools Table, Materials and Methods, Figures, Data Availability Section)
If study protocol has been **pre-registered, provide DOI in the manuscript**. For clinical trials, provide the trial registration number **OR** cite DOI.	Not Applicable	
Report the **clinical trial registration number** (at ClinicalTrials.gov or equivalent), where applicable.	Not Applicable	

Laboratory protocol	Information included in the manuscript?	In which section is the information available? (Reagents and Tools Table, Materials and Methods, Figures, Data Availability Section)
Provide DOI OR other citation details if **external detailed step-by-step protocols** are available.	Yes	Material and Methods

Experimental study design and statistics	Information included in the manuscript?	In which section is the information available? (Reagents and Tools Table, Materials and Methods, Figures, Data Availability Section)
Include a statement about **sample size** estimate even if no statistical methods were used.	Not Applicable	
Were any steps taken to minimize the effects of subjective bias when allocating animals/samples to treatment (e.g. **randomization procedure**)? If yes, have they been described?	Not Applicable	
Include a statement about **blinding** even if no blinding was done.	Not Applicable	
Describe **inclusion/exclusion criteria** if samples or animals were excluded from the analysis. Were the criteria pre-established?<br><br>If sample or data points were omitted from analysis, report if this was due to attrition or intentional exclusion and provide justification.	Not Applicable	
For every figure, are **statistical tests** justified as appropriate? Do the data meet the assumptions of the tests (e.g., normal distribution)? Describe any methods used to assess it. Is there an estimate of variation within each group of data? Is the variance similar between the groups that are being statistically compared?	Yes	Material and Methods

Sample definition and in-laboratory replication	Information included in the manuscript?	In which section is the information available? (Reagents and Tools Table, Materials and Methods, Figures, Data Availability Section)
In the figure legends: state number of times the experiment was **replicated** in laboratory.	Yes	Figures
In the figure legends: define whether data describe **technical or biological replicates**.	Yes	Figures

Ethics

Ethics	Information included in the manuscript?	In which section is the information available? (Reagents and Tools Table, Materials and Methods, Figures, Data Availability Section)
Studies involving **human participants**: State details of **authority granting ethics approval** (IRB or equivalent committee(s), provide reference number for approval.	Not Applicable	
Studies involving **human participants**: Include a statement confirming that **informed consent** was obtained from all subjects and that the experiments conformed to the principles set out in the WMA Declaration of Helsinki and the Department of Health and Human Services Belmont Report.	Not Applicable	
Studies involving **human participants:** For publication of **patient photos**, include a statement confirming that consent to publish was obtained.	Not Applicable	
Studies involving experimental **animals**: State details of **authority granting ethics approva**l (IRB or equivalent committee(s), provide reference number for approval. Include a statement of compliance with ethical regulations.	Not Applicable	
Studies involving **specimen and field samples:** State if relevant **permits** obtained, provide details of authority approving study; if none were required, explain why.	Not Applicable	

Dual Use Research of Concern (DURC)	Information included in the manuscript?	In which section is the information available? (Reagents and Tools Table, Materials and Methods, Figures, Data Availability Section)
Could your study fall under dual use research restrictions? Please check biosecurity documents and list of **select agents and toxins** (CDC): https://www.selectagents.gov/sat/list.htm	Not Applicable	
If you used a select agent, is the security level of the lab appropriate and reported in the manuscript?	Not Applicable	
If a study is subject to dual use research of concern regulations, is the name of the **authority granting approval and reference number** for the regulatory approval provided in the manuscript?	Not Applicable	

Reporting

The MDAR framework recommends adoption of discipline-specific guidelines, established and endorsed through community initiatives. Journals have their own policy about requiring specific guidelines and recommendations to complement MDAR.

Adherence to community standards	Information included in the manuscript?	In which section is the information available? (Reagents and Tools Table, Materials and Methods, Figures, Data Availability Section)
State if relevant guidelines or checklists (e.g., **ICMJE, MIBBI, ARRIVE, PRISMA**) have been followed or provided.	Not Applicable	
For **tumor marker prognostic studies,** we recommend that you follow the **REMARK** reporting guidelines (see link list at top right). See author guidelines, under 'Reporting Guidelines'. Please confirm you have followed these guidelines.	Not Applicable	
For **phase II and III randomized controlled trials**, please refer to the **CONSORT** flow diagram (see link list at top right) and submit the CONSORT checklist (see link list at top right) with your submission. See author guidelines, under 'Reporting Guidelines'. Please confirm you have submitted this list.	Not Applicable	

Data Availability

Data availability	Information included in the manuscript?	In which section is the information available? (Reagents and Tools Table, Materials and Methods, Figures, Data Availability Section)
Have **primary datasets** been deposited according to the journal's guidelines (see 'Data Deposition' section) and the respective accession numbers provided in the Data Availability Section?	Yes	Data Availability Section
Were **human clinical and genomic datasets** deposited in a public access-controlled repository in accordance to ethical obligations to the patients and to the applicable consent agreement?	Not Applicable	
Are **computational models** that are central and integral to a study available without restrictions in a machine-readable form? Were the relevant accession numbers or links provided?	Not Applicable	
If publicly available data were reused, provide the respective **data citations in the reference list**.	Yes	Material and Methods; reference list
